# Therapeutic Affordances of Social Media: Emergent Themes From a Global Online Survey of People With Chronic Pain

**DOI:** 10.2196/jmir.3494

**Published:** 2014-12-22

**Authors:** Mark Merolli, Kathleen Gray, Fernando Martin-Sanchez

**Affiliations:** ^1^Health and Biomedical Informatics CentreThe University of MelbourneMelbourneAustralia

**Keywords:** social media, chronic disease, chronic pain, therapeutic affordances, thematic content analysis, patient-reported outcomes, model

## Abstract

**Background:**

Research continues to present tenuous suggestions that social media is well suited to enhance management of chronic disease and improve health outcomes. Various studies have presented qualitative reports of health outcomes from social media use and have examined discourse and communication themes occurring through different social media. However, there is an absence of published studies examining and unpacking the underlying therapeutic mechanisms driving social media’s effects.

**Objective:**

This paper presents a qualitative analysis thoroughly describing what social media therapeutically affords people living with chronic pain who are self-managing their condition. From this therapeutic affordance perspective, we aim to formulate a preliminary conceptual model aimed at better understanding "how" social media can influence patient outcomes.

**Methods:**

In total, 218 people with chronic pain (PWCP) completed an online survey, investigating patient-reported outcomes (PROs) from social media use. Supplementary to quantitative data collected, participants were also given the opportunity to provide further open commentary regarding their use of social media as part of chronic pain management; 68/218 unique users (31.2%) chose to provide these free-text responses. Through thematic content analysis, 117 free-text responses regarding 10 types of social media were coded. Quotes were extracted and tabulated based on therapeutic affordances that we had previously identified. Inductive analysis was then performed to code defining language and emergent themes central to describing each affordance. Three investigators examined the responses, developed the coding scheme, and applied the coding to the data.

**Results:**

We extracted 155 quotes from 117 free-text responses. The largest source of quotes came from social network site users (78/155, 50.3%). Analysis of component language used to describe the aforementioned affordances and emergent themes resulted in a final revision and renaming of therapeutic affordances: "exploration" (52/155, 33.5% of quotes), "connection" (50/155, 32.3% of quotes), "narration" (33/155, 21.3% of quotes), "adaptation" (13/155, 8.4% of quotes), and "self-presentation" (7/155, 4.5% of quotes). Of the most described affordances, "exploration" was based on a propensity for participants to explain their social media use for information seeking purposes. "Connection" placed greater emphasis on interaction, highlighting themes of "exchanging information" and "mitigating isolation". Responses regarding "narration" highlighted the value of shared experiences and the emotionally cathartic role this plays.

**Conclusions:**

Much of the efficacy of social media may be explicable via a closer examination of therapeutic affordances. Particular areas that warrant attention include social media’s ability to filter and guide people to useful information, connect individuals, and share experiences. Further research into a variety of chronic conditions is warranted. Coupled with the results of the present study, a greater theoretical basis detailing how social media may foster health outcomes may lead to an improved evidence base for conducting research and may inform recommendations for social media use in chronic disease management.

##  Introduction

### Background

Studies continue into the therapeutic effects of social media use in chronic disease management, suggesting that social media may be beneficial to people living with chronic disease [1-3]. However, we have not yet gained a detailed knowledge of these effects. Common research approaches used in qualitative studies in the social media domain include, but are not exclusive to, examining the data mined from social network sites (SNS) and online support groups [4,5], thematic analysis of blog content [6], and phenomenological coding of video narratives on YouTube [7,8]. Results commonly describe themes of support, information provision, and online privacy concerns. However, there is relatively little published literature that contextualizes such themes within a model for understanding what underlying mechanisms drive observed effects and health outcomes from social media use.

Previous studies in psychology have examined the relationship between individuals and their environment in an attempt to clarify how interaction may explain the ensuing behaviors of individuals [9]. The theory of “affordances” describes this relationship, which we discuss further in the following section. When interaction with social media mediates health outcomes, the affordance concept can be adapted to examine the underpinning properties of various social platforms affording therapeutic effects. Henceforth, our study refers to “therapeutic affordances” to present an in-depth analysis of such factors. Reference to therapeutic affordances within a health context has previously been seen in only a few studies. Examples include neurological dysfunction and mental health [10-12]. However, the term was contextualized differently. Fetters and Ellis proposed that therapeutic affordances guide action as they “give insight into the relationship between the patient’s altered person constraints and the successful accomplishment of meaningful goals” (p. 145) [10].

### The Theory of Affordances

Affordance was originally coined by psychologist James Gibson in relation to his work in visual perception. He used the term to describe environmental properties relative to individuals. More technically, affordances are actionable possibilities that exist within the relationship between the actor and features of the environment [13,14]. Donald Norman takes this to a more concrete level, bringing affordance theory into mainstream culture, through its application to the design of everyday things [15]. He stresses the importance of both the actionable possibility and the way that this is conveyed or made visible to the actor [13]. This refined model of affordance leans heavily on the user’s experiences, understanding, goals, and past experiences, rather than purely the physical qualities of the environment or object as suggested by Gibson [15]. Hence, Norman suggests that when designing something, every attempt should be made to convey information that intimately outlines the affordance and highlights trouble-free use [15].

### Therapeutic Affordances of Social Media

Affordances have been discussed in various academic articles in different contexts [13,16,17]. However, the way they are described and related back to the work of Gibson and Norman differs considerably. Relative to social media, appropriations of affordances have more recently evolved from human-computer interaction (HCI) research. HCI researchers began using the term “affordances” when studying computer technologies as an artifact. In reference to the present study, social media becomes the artifact. Affordances have included physical affordances (supporting or facilitating physical activity), cognitive (supporting thinking or learning models), sensory (supporting or facilitating sensing), functional (supporting physical activity for a functional purpose), and motivational (supporting motivational needs) [13]. To describe the likely end uses and effects of social media, the focus is placed on the platform, its interface, and the formally described services it provides. Much of the utility of describing affordances of social media in HCI research relates to usability factors because fundamentally, affordances of technology are emergent properties of the integration of the user and the medium [18,19]. However, definitions of social media refer to them as tools designed to allow for the realization of the dynamic interactive qualities of Web 2.0, namely openness, participation, interaction, and collaboration [1,20-22]. Therefore, a simple examination of usability via interaction between user, hardware, and platform interface is not sufficient [13]. In the case of social media as the artifact, it is the user who is ultimately given importance because it is their preferences, attitudes, beliefs, intentions, motivations, experiences, and needs that all converge to allow therapeutic affordances to be realized [13].

Relative to health outcomes, thought needs to go into how social media are being used and in what context. Therefore, one of the preliminary tasks is to identify the type of user being targeted and the environment in which social media are to be used. This provides insight into the user’s motivations for using social media. In the present study, the context is people with chronic pain (PWCP) self-managing their condition. Referring to Norman’s approach to affordances is thus helpful in this context, as it suggests that different users will approach, understand, and react to the same object differently [15].

### Aim

Building on the foundation of an extensive literature review [23], the aim of this paper is to understand how a more targeted approach to social media use by considering therapeutic affordances may better address the needs of people living with and self-managing chronic disease. We theorize that different social media interactions can precipitate different health effects for different people. We anticipate that this may guide clinicians’ decision making surrounding whether social media may form a meaningful part of patient management and also may guide further research in this field.

## Methods

### Overview

**Figure 1:**
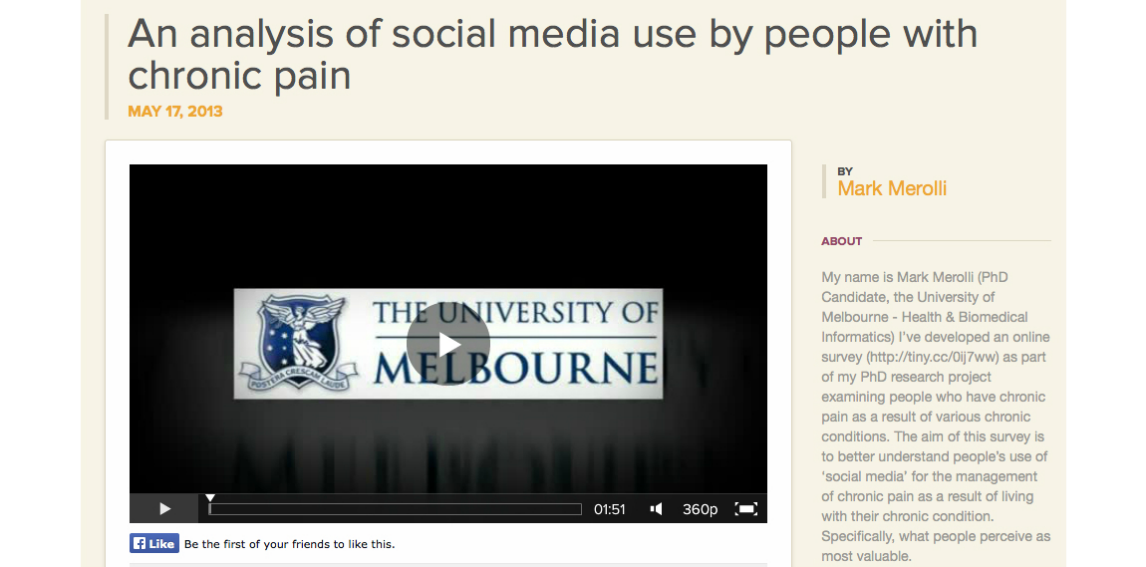
Screenshot from chronic pain online survey recruitment video (prepared with Animoto).

This paper reports on findings from the analysis of qualitative data collected as part of a global online survey of PWCP, investigating patient-reported outcomes (PROs) from their social media use to manage chronic pain (Figure 1). This follows approaches to developing and testing conceptual models in health [24]. The Human Research Ethics Committee at the University of Melbourne approved this study (ID No. 1339414).

### Rationale for Qualitative Study

Previous research on social-mediating technologies in health has employed thematic content analysis (TCA) to examine qualitative data [6-8,25-29]. Data are collected from a given set of participants reflecting on their experiences within a particular area of study to identify themes [30,31]. The researcher’s epistemological stance is an objective one, where themes are extrapolated from the data in an attempt to give meaning to the commonality of collective participants [30]. These data can be coded “inductively”, meaning themes are pulled from the data gradually, or “deductively”, whereby themes are imposed on the data at the outset as a way of approaching it [31]. When conducting inductive coding, grounded theory is used to describe the process whereby categories are identified as they emerge from the data [31]. These themes are given names or labels in a manner that reflects the words of the participants as a whole. In reality, in the realm of phenomenological research, thematic content analysis requires the researcher to identify meaning within themes and then further put this into the context of the study. The TCA process is not a complete analysis until this has occurred. While open to more interpretative bias, TCA adds another dimension to health research, particularly research with substantial social components. Hence, it is a good fit for the present study. Unlike quantitative methods, which can still be applied to qualitative data, TCA allows the findings to be viewed from the participant’s perspective, in their own words. This is particularly useful when investigating a complex construct, such as social media [31].

### Recruitment and Data Collection

Adults (18 years or older), with chronic pain (3 months or greater), who used social media as part of their self-management were invited to participate in this online survey via online social networks. Searching Google periodically from March 1 through to May 20, 2013, terms such as “online health networks”, “online pain support communities”, “chronic pain organizations”, “chronic disease organizations”, and “international pain organizations” were used to identify potential recruitment channels. Searching was limited to English language. Also included were common social networks, and we targeted active chronic pain groups, such as those on Facebook, Twitter, Daily Strength, and PatientsLikeMe. We also contacted various other influencers (at the support group/organizational level and individual level based on word of mouth).

Requests for assistance were emailed to each organization/group’s moderator. We made it clear that we were focusing the survey on “pain interference” as a result of living with chronic disease. The email contained a link directly to the survey, with the plain language statement and informed consent appearing on the survey webpage. This allowed moderators to review the suitability of the research to their members. A recruitment video was also created by the study’s primary investigator to complement the email, and a link to the video was pasted into the email text [32]. If the moderator was willing to post the survey to their members, a link to the survey was placed on the websites of the groups, shared on social media, and included in newsletters where appropriate. This emerging approach to recruitment using social media is documented in the literature [33-36] and was the subject of an article published elsewhere [37]. Given that we relied on a viral dissemination of the survey link via social media and did not invite participants directly via individual emails, it was impossible to calculate response rate based on number of invitations compared to responses.

The survey was conducted using the online survey software, SurveyMonkey. The survey was open from May 21 to June 30, 2013. Participants were asked to provide quantitative and qualitative data in a variety of areas: demographics, health/pain status, social media used, therapeutic affordances, and PROs from use. The full survey instrument can be found in Multimedia Appendix 1.

The focus here is on free-text responses from this survey. Supplementary to quantitative data collected (which is the focus of a separate paper), participants were given opportunities at several points within the survey to provide open commentary relating to the social media platforms they used as part of chronic pain management. The social media they could comment on included SNS, blogs, wikis, microblogs, virtual worlds, tagging/aggregation sites, video sharing sites, photo sharing sites, chat rooms, and discussion forums. An example of how the open commentary questions were phrased was “Please use this space if you would like to comment further about your use of BLOGS for your chronic pain self-management”.

### Participants

In total, 231 PWCP consented to take part in the survey; 4 of these supplied no further information and a further 9 answered “no” to the question, “Do you have chronic pain?” The final dataset thus represented 218 completed surveys. A subset of all participants (68/218, 31.2%) chose to provide free-text responses. Table 1 shows their demographic characteristics. The data highlight a majority of married/partnered females, not working because of ill health. However, a wide range of ages and education levels are represented.

**Table 1:** Participant demographics (n=68).

Characteristics		n (%)
**Gender**		
	Male	10 (15)
	Female	58 (85)
**Age range**		
	18-29	14 (21)
	30-39	14 (21)
	40-49	16 (24)
	50-59	16 (24)
	60+	8 (12)
**Marital status**		
	Never married	17 (25)
	Currently married/partnered	41 (60)
	Separated/divorced/widowed	10 (15)
**Level of education**		
	High school or less	25 (37)
	College/University completed	26 (38)
	Post-graduate degree completed	17 (25)
**If not working for pay (reason?) (n=40)**		
	Ill health	34 (85)
	Other	6 (15)

### Data Analysis

#### Dataset

The dataset for analysis was a total of 117 free-text responses given by these 68 participants; 155 separate quotes were extracted. Each quote remained linked to the respondent with a unique identifier. The quotes pertained to 10 different types of social media. SNS accounted for the majority of quotes (78/155, 50.3%), followed by much smaller samples. The full list can be seen in Table 2.

**Table 2:** Dataset based on platform.

Platform	Used each platform (N=218), n	Free-text responses collected, n	Supplying free-text responses, n (%)	Extracted quotes, n	Total quotes (N=155), n (%)
Social network sites	189	58	58/189 (31)	78	78 (50.3)
Discussion forums	86	18	18/86 (21)	16	16 (10.3)
Blogs	88	11	11/88 (13)	14	14 (9.0)
Wikis	74	10	10/74 (14)	10	10 (6.5)
Videosharing sites	60	7	7/60 (12)	10	10 (6.5)
Photosharing sites	18	5	5/18 (28)	9	9 (5.8)
Microblogs	29	6	6/29 (21)	6	6 (3.9)
Chat rooms	11	3	3/11 (27)	5	5 (3.2)
Virtual worlds	7	2	2/7 (29)	4	4 (2.6)
Tag/Aggregators	12	2	2/12 (17)	1	1 (0.6)

#### Procedure

**Figure 2:**
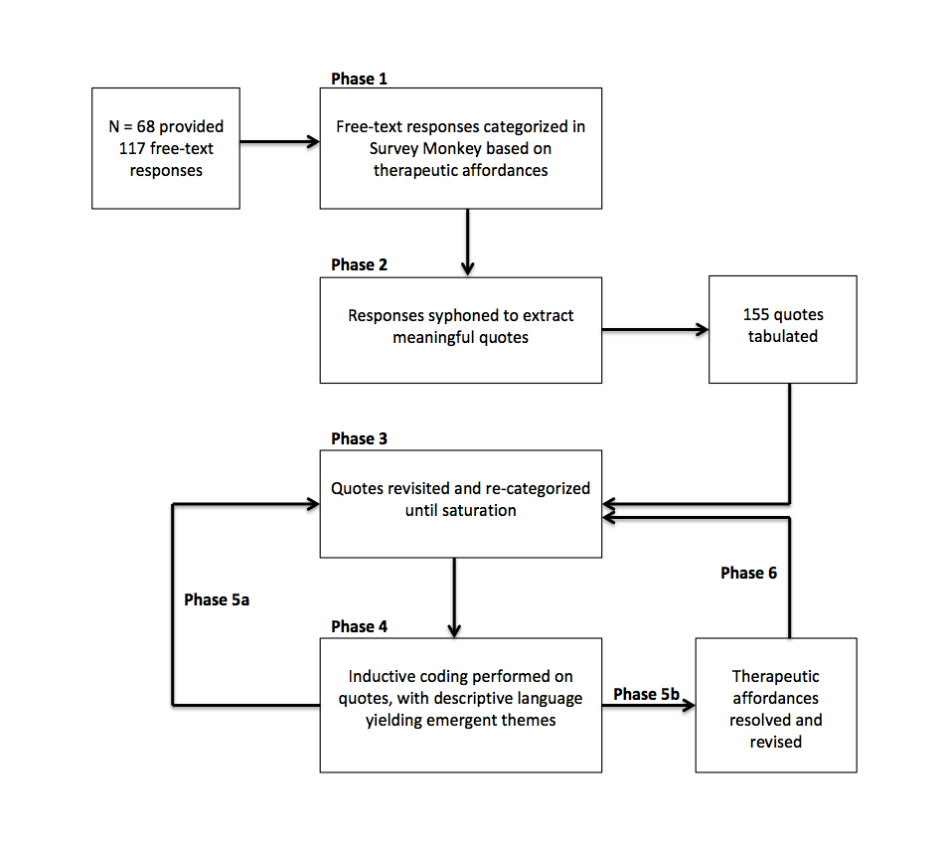
Study workflow.

Next, 3 investigators used TCA to examine each quote, developed the coding scheme, and applied the coding to the data. Initially, quotes were coded deductively according to their best fit to a set of therapeutic affordances of social media that we had previously identified: identity, flexibility, structure, narration, and adaptation [23]. Then, an alternative inductive analysis was performed to group quotes into other possible themes more closely based on the language components of the quote. Following this two-step coding process, we reviewed and renamed our previous set of therapeutic affordances. A step-by-step workflow can be seen in Figure 2.

In Phase 1, using the “analyze data” function in SurveyMonkey, the free-text responses for each platform were accessed. The first step was to calculate the number of responses provided. The next step in this analysis was to read each response individually and create preliminary categories in SurveyMonkey based on the therapeutic affordances: identity, flexibility, structure, narration, and adaption. Any responses containing quotes referring to two or more therapeutic affordances were categorized as “mixed”. The coding procedure was challenged by responses that appeared to be between categories and those containing descriptions that were outside the proposed categories.

In Phase 2*,* based on the affordances applied in Phase 1, responses were syphoned to extract meaningful quotations that pertained to the respective therapeutic affordance. In several instances, responses contained more than one quote spanning more than one affordance. The quotes were then tabulated to create a better visualization of the data (Multimedia Appendix 2). A picture emerged of the prevalence of each affordance described by participants. Quotes were tabulated according to therapeutic affordance, social media type, and whether the connotation was positive or negative.

In Phase 3, all quotes were revisited several times to ensure consistency of categorization. This process was repeated until no further re-categorization was necessary.

In Phase 4, the coding method was altered, focusing on inductively analyzing the data. Descriptive language in each quote was used to help formulate themes emergent from within the data that captured the essence of what the quote described. This process was performed independently by all 3 researchers and repeated until no new themes emerged. Themes were discussed and compared for overlap. Any consistent themes were automatically coded. This process yielded 15 themes that captured the essence of each therapeutic affordance.

In Phase 5, the entire dataset was revisited (5a). The descriptive language and themes coded from the quotes describing each affordance in Phase 4 were reconsidered and grouped, thus resolving into a revised set of 5 therapeutic affordances: self-presentation, connection, exploration, narration, and adaptation (5b).

The process in Phase 5 also helped to code those quotes that did not fit neatly within one category or those that initially appeared to span multiple categories. Thus, Phase 3 was once again revisited until Phase 6, which revalidated the newly coined affordances, was complete.

## Results

### Therapeutic Affordances Described

#### Overview

Based on the 155 extracted quotes, responses were mostly positive (134/155, 86.5%) (Multimedia Appendix 2). The five finalized therapeutic affordances appeared in the data to varying degrees. The largest number of quotes related to the “exploration” (52/155, 33.5%) and “connection” (50/155, 32.3%) affordances, followed by “narration” (33/155, 21.3%), then “adaptation” (13/155, 8.4%), and “self-presentation” (7/155, 4.5%). Key language used by participants to describe each affordance and the inductively identified themes are presented and discussed in Table 3. The revised therapeutic affordances are then presented. The most representative quotes were extracted and examples highlighted to demonstrate the themes in question.

**Table 3:** Descriptive language, inductive themes, and validated therapeutic affordances.

Language	Theme	Therapeutic affordance
Disclosure	Disclosure	Self-presentation
Control	Control	
Private	Identification	
Anonymity		
Masquerade		
Communicate	Interaction	Connection
Exchange	Exchanging information	
Share	Support	
Discuss	Mitigating isolation	
Advice	Geographic freedom	
Support		
Isolation		
Alone		
Worldwide		
Look	Information seeking	Exploration
Research	Learning	
Find	Reputability	
Learn		
Monitor		
Moderate		
Share	Imparting knowledge	Narration
Stories	Understanding	
Experiences	Emotional catharsis	
Journey		
Distress		
Flared-up	Variation in use	Adaptation
Affected		
Bad		
Sick		
Hospital		

#### Self-Presentation: Disclosure, Control, and Identification

This affordance was present in the responses participants gave regarding the level of information they presented to the world via social media. It also highlighted user preferences surrounding identifiable versus anonymous interactions. Participants did not appear to place a great emphasis on the differing self-presentations afforded by social media. However, the quotes obtained showed a relatively equal share of positive and negative perceptions. Information disclosure was a topical theme discussed in terms of self-presentation. Participants showed a propensity to value private interaction and to limit the exposure of their online activity: “The discussion forum we use is a private support group, so only other sufferers of my condition are able to participate” [resp. 204].

This was reiterated more strongly in one person’s perception of potentially negative consequences from disclosing personal information. Powerful language included words like “fear” and “masquerading”. This same participant described the apprehension attached to personal disclosure. They felt strongly about their personal information being used against them: “My fears around disclosure and how it impacts psychologically physically and emotionally is enough to prevent me from disclosing in the social media” [resp. 64] and “I do not want to disclose my personal and painful journey via a social network site for it to be highlighted by others and ‘used’ as a way to finish me in my job” [resp. 64].

However, positive opinion describing “self-presentation” contained a sense of control and autonomy found in social media use: “This is an excellent medium for me to be able to control my social interactions” [resp. 120], “Social network sites allow me to interact with others on my own terms” [resp. 184], and “writing a decent-ish blog can allow you to be the person you were to an extent” [resp. 229].

Participants had differing preferences towards whether others knew their identity. In one instance, one participant described a desire to remain anonymous to friends and family, yet known to other PWCP: “I find these boards are more intimate if I can remain anonymous to family and friends but known to others on the board” [resp. 176].

Conversely, anonymity was also used to highlight the potential hazards surrounding self-presentation and an inability to verify identity through social media: “I see individuals masquerading as members of the medical profession” [resp. 191].

#### Connection: Interaction, Exchanging Information, Support, Mitigating Isolation, and Geographic Freedom

An ability to connect with others featured frequently in the responses. Participants commonly used social media to reach out to others in similar situations, share/exchange information, and offer support. A key objective was mitigating isolation. This was aided by a sense of freedom afforded by social media to interact when and where it suits.

Connection to others was one activity particularly suited to social media. Participants commonly described their interactions enabled by social media: “We are able to help each other with practical advice about drug side effects etc” [resp. 83], “Having immediate access to information and people is enlightening” [resp. 206], and “Talking to people who understand is great” [resp. 180].

Participants reported actively sharing and exchanging information. The words “sharing” and “exchanging” of information appeared frequently throughout the responses, with words like “valuable” often used in conjunction. Sharing of information ranged from general disease-specific information and advice, through to practical tips. On a more personal level, participants tied the ability to exchange information to an enhanced feeling of self-worth and validation, often inhibited by living with chronic illness: “I feel valued sharing my resources with others” [resp. 79], “I focus on sharing what knowledge or information I have in other areas, this mitigates the constant feeling of failure that comes along with ill health and the inability to be more active” [resp. 177], and “The exchange of information available is invaluable” [resp. 38].

Like all therapeutic affordances, connection seen via information exchange is not immune to criticism. One person who pondered the reputability of information exchanges expressed this as “Unmoderated sites are potentially dangerous, unqualified ppl routinely diagnose, recommend treatment, use research findings inappropriately, misinform other ppl who may not know any better” [resp. 8].

Providing and receiving support was described as another valuable utility of social media. A sense of camaraderie enabled by social media was outlined, bringing together people in similar situations. Support fostered through interactions was emotionally cathartic to participants and gave them the motivation to persist with management: “only a person with similar problems really gets what you’re going through” [resp. 201], “we all support each other during our tough times and still communicate even during our good days” [resp. 140], and “Being able to communicate with people can be up lifting and cathartic” [resp. 229].

The strength of social media to connect was evident as analysis uncovered strong positive sentiments towards social media use as part of chronic pain management: “I find general support from others with the same condition on these sites has stopped my suicide attempts” [resp. 217], “[social network sites] actually saved my life. The people I connected with were other with MS…we all support each other” [resp. 140], and “The friendships and information I’ve gained from those have been life saving—probably in the literal sense” [resp. 71].

On the other hand (and although only a small representation), those who perceived “connection” negatively discussed a sense of withdrawal and frustration: “Social networking can sometimes have a negative impact ie withdrawal from real life” [resp. 56] and “Frustrating and at times more painful trying to explain to strangers” [resp. 124].

Mitigating isolation was the most present theme describing “connection”, accounting for 28% (14/50) of quotes. Participants felt extremely positive towards social media’s ability to keep them from being dominated by loneliness. Feeling connected and knowing they were not alone were apparent effects. The words “alone” and “isolation” appeared six and eight times respectively: “Without social networking I would feel far more alone when my conditions are bad” [resp. 46], “pain is isolating in real life and social networking can help to reduce the amount of isolation” [resp. 56], and “Social network sites have allowed me to have a social life…when the pain is bad, which is frequent, I cannot leave my house and spend time with friends” [resp. 196].

A final element underlying “connection” was seen through the abundant quotes detailing reach and spread of communication afforded by social media. Participants freely commented that their use of social media provided borderless interactions, thus highlighting that managing chronic disease can span the world. Social media allows these people the opportunity to extend their networks: “[SNS] is often putting patients in touch with treating practitioners around the world” [resp. 79], “people all over the world can communicate and support others in their same situation” [resp. 78], and “I live in a regional area and helpful Dr. services are not available! I’m discovering the positives of using the sites” [resp. 164].

Geographic freedom has also increased the availability of support. Being able to connect with fellow PWCP internationally means that time of day need no longer be a hurdle: “now I have friends worldwide…they are there day or night if I need” [resp. 71] and “are mostly helpful due to the ‘you are not alone’ value” [resp. 215].

#### Exploration: Information Seeking, Learning, and Reputability

“Exploration” was the most noted therapeutic affordance based on number of quotes. Survey participants frequently described searching for information. A variety of search-related activities were reported

Above all, participants described using social media for guidance towards useful information for pain management. At times the search for information yielded resources that filtered further useful resources. Participants reported using social media to seek out information on a wide variety of matters relevant to their condition. This included disease-specific information, research, and treatments: “I selectively filter through information...I look for information around the management of pain and adapting in certain situations” [resp. 51], “The blogs of health researchers and professionals are very important tools for finding new information, current research etc” [resp. 217], and “Practical tips, medication, treatment recommendations have often lead me to helpful sites” [resp. 190].

Some participants valued the information found through social media: “Gives me more information about a specific condition or more things to discuss with my doctor” [resp. 200]. Again, some voiced cautions. Using the example of wikis, two participants highlighted: “Wikis are not always particularly useful, as entries tend to be very general and not always up to date” [resp. 190] and “Seem to be most prone to bias…so bad information doesn’t get corrected” [resp. 125].

Another process was that of “learning” resulting from use. Participants declared that search and guidance promoted by social media were useful to aid learning about managing pain, disease-specific knowledge, and self-management: “Have particularly used social networking to learn tips about managing/avoiding pain” [resp. 112], “…use blogs so as I can learn as much as I can about my condition. Knowledge is power, etc” [resp. 227], and “They have definitely helped my understanding of nerve pain and pain in general. I have grasped anatomical and medical info much easier with YouTube” [resp. 229].

From a more cautious perspective, quality of information resonated with some survey participants: “Value depends on quality of information” [resp. 190]. Participants consider this pivotal to the success of self- management via social media. One participant described the free flow of information made available by social media: “The beauty of social media is that there is no censor or control so the right information gets through” [resp. 78].

However, this conflicts somewhat with the perceptions that information made available via social media can at times be disreputable: “…can be misleading depending on who has written the article” [resp. 66] and “Seem to be most prone to bias...so bad information doesn’t get corrected” [resp. 125].

This may be addressed via careful consideration of individual preferences for moderated and monitored interactions, as seen in many quotes: “I much prefer a medically knowledgeable, balanced platform such as a monitored [one] where good, sensible advice and help is offered” [resp. 40] and “one becomes discriminating with the types of boards and if the moderator is a good one, this sets the tone of the board and generally adds to the value of the information” [resp. 190].

#### Narration: Imparting Knowledge, Understanding, and Emotional Catharsis

The narrative affordance was well documented in the results of this survey. Multiple instances of the positive effect of sharing experiences via social media were described. This was true of both sharing one’s own experiences and accessing another’s experiences. Accessing other’s experiences was most notable, accounting for 48.5% (16/33) of total “narration” quotes.

Where more active participation was described, imparting knowledge by sharing one’s own experiences with chronic pain fostered a sense of personal satisfaction and self-worth. This made participants feel useful, as they were able to impart knowledge they had accumulated: “networking sites make me feel useful as I can share my stories with newly diagnosed people” [resp. 185], “I write reflective and creative pieces about my journey in general...often I won’t even discuss the physical side of it, but rather the lessons learned” [resp. 189], and “It is very helpful to share stories, symptoms, and problems with other sufferers” [resp. 223].

Participants also described that accessing the experiences of others played a beneficial role in improving understanding. Through accessing experiences and narratives about pain from other people, participants felt able to better manage their condition: “I find it helpful to know how other people manage their own pain” [resp. 191] and “having people who can make comments and talk about their stories give me more information about what other people are dealing with” [resp. 32].

However, narration was most commonly discussed as a means to manage the emotional burden of living with chronic pain. Participants discussed the catharsis, comfort, and validation from accessing other people’s experiences: “It just helps to know there are other people in the same boat who work hard at their disorder and succeed. It gives me hope and patience to keep trying” [resp. 212], “Seeing others who are like me, is a HUGE comfort” [resp. 190], and “Many of my own experiences were also shared by others. Often it is quite validating to see others have been thru the same things I have” [resp. 190].

Conversely, some did caution that the narrative experience could be counterproductive, as people may be potentially distressed from sharing experiences: “I do not feel able to share my experience as yet as I become very distressed when I think/write/talk about my condition” [resp. 47], “Sometimes reading other peoples journeys is useful but I do not like to immerse myself in too many down stories as it makes me worse so I am careful about what I read” [resp. 209], and “I find most blogs very distressing as I ‘see’ how my future may progress” [resp. 51].

One participant took this further and described the potential to become alienated by sharing experiences online: “Unfortunately it can often feel that when people read your posts in regard to chronic pain, it can create speculation about the condition...it can make me feel, especially being a young male, that I am just a whinger and need to toughen up” [resp. 84].

In a similar light, narratives may also provide greater scope to malinger and catastrophize one’s condition: “I do find many social network chronic pain sites regular participants too needy. I can’t abide the constant call for, supply of prayers and the desperation for others to feel/ understand/ get their pain” [resp. 40] and “sites become a platform for particular ppl to use to verbalize complaints and negativity, without any constructive discourse” [resp. 8].

#### Adaptation: Variation in Use

The “adaptation” affordance captures the way social media enables participants to adapt their self-management behaviors in relation to their condition status and/or needs at particular points in time in various ways. Most apparent was social media’s utility in allowing participants to alter their self-management when pain flared or health deteriorated: “I am severely affected at present. I cannot speak, or tolerate the stimulation of people being around me...I am bedridden and need to be in a very low stimulus environment…but youtube is ok for me with care” [resp. 190], “It’s nice to be able to learn even when I can’t read or sit up” [resp. 27], and “I will post a photo on Instagram if I am in hospital and people want to know how I am going but I am too sick to tell them” [resp. 189].

##  Discussion

### Principal Findings

#### Refining Therapeutic Affordances

In our previous work, we postulated five therapeutic affordances: “Identity” was used to present perceptions regarding disclosure of identity in online social environments. “Flexibility” described the time-space freedom enabled by social media, such as the ability to interact at a time suiting the individual and wherever one chooses. “Structure” described the guidance and filtration present in information seeking that social media can provide. “Narration” encompassed social media’s utility to provide a platform to share stories of illness, and finally, “adaptation” referred to the capacity for one’s self-management to evolve through social media to meet particular needs based on current disease status [23].

While our initial nomination of these affordances provided a useful frame for early theorizing, analysis of the qualitative survey data enabled a much richer conceptualization and categorization of the underlying themes describing each therapeutic affordance. Bearing in mind that the use of the term affordance has previously attempted to clarify behaviors an individual may perform, referring to affordances as all actionable possibilities latent in the environment [9], we defined two tests of our affordances in relation to the data from the present study: Does the therapeutic affordance account for the data? Does the therapeutic affordance describe an actionable possibility? Consequently, the data highlighted a lack of clarity in some of our early descriptions. “Narration” and “adaptation” passed the tests; however, the other three affordances were found to be incongruent.

First, the label “identity” is not an actionable term, nor does it describe elements of disclosure or privacy central in participant responses. Quotes described preferences for anonymity, control of one’s self-image, the precautions required when interacting online and not being able to verify identity. Therefore, in regards to all of these elements relevant to impression management, “self-presentation” is more descriptive and appropriate than “identity” [38].

Social media are often praised for the “flexibility” they offer health consumers to interact where and when they choose. Responses in the present study also displayed a propensity to praise the opportunity to connect and interact with others (including borderless communication). However, participants also valued supporting one another, mitigating isolation, and exchanging information. Descriptive language was slanted towards communication, discussion, and exchange rather than the flexibility afforded by social media. Thus, the therapeutic affordance is better described as the “connection” of individuals.

“Structure” presented the same semantic challenge. While one can use “structure” as a verb, it is ambiguous. Previous notions that social media guide and filter information seeking still persist. However, the overarching theme from the survey data was “information seeking”. Participants used language such as “finding”, “looking”, and “searching” for information. Hence, “exploration” is more appropriate.

Interaction and sharing seen in “connection” and “narration” embody the very participatory and collaborative nature on which Web 1.0 has evolved into Web 2.0 [1,39]. However, despite “exploration” being the most discussed afforded use of social media (33.5% of total responses), it is important to recognize the language used to describe it (ie, seeking, finding, searching, looking). The language used is information-focused, not user-centered [40,41]. This sentiment has been previously reported [3], indicating that perhaps social media are still not being realized to their full potential as many-to-many communication tools.

Similar difficulties surrounding labeling affordances of social media are apparent in other academic literature. In a study of social media affordances within organizational processes [42], four affordances are based on the authors’ perceptions of the activities performed in business processes salient to social media’s utilities: visibility, editability, persistence, and association. We suggest that ideas such as “visible” and “persistent”, while informative, are contentious in terms of whether they are indeed actionable by people or rather, characteristic of content. These contentions warrant attention by all researchers working with the concept of social media affordances.

#### Proposing a Theoretical Model

**Figure 3:**
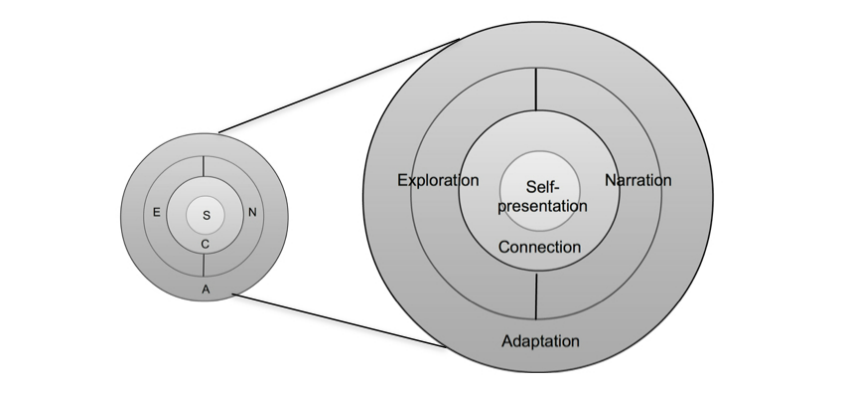
SCENA model of therapeutic affordances of social media.

Resulting from this reflection on the analysis presented in this paper, the therapeutic affordances of social media were refined and resolved into self-presentation, connection, exploration, narration and adaptation (SCENA). This resolution enabled us to develop a conceptualization of these affordances relative to health outcomes, not only revising their definitions but also relating them to each other within a new framework that can be visualized as a theoretical model (Figure 3).

The SCENA Model of Therapeutic Affordances of Social Media has at its core preferences and perceptions regarding one’s online image or digital identity. “Self-presentation” feeds into the ability of social media to “connect” individuals. The next layer is shared by “exploration” and “narration”, both of which take into consideration varying preferences for self-presentation and how individuals connect. The outermost layer of the model is “adaptation”, that is, social media use allowing self-management behaviors to adapt to individual needs at given points in time. This will influence and be influenced by the other therapeutic affordances to varying degrees.

#### Theorizing About What Makes Therapeutic Affordances Effective

Different categorizations have been proposed as ways to organize affordances of social media. If therapeutic affordances are to be meaningful in a chronic disease management or broader health context, then categorizing them to show how they function to produce effects is a critical contribution that health informatics can make.

The use of classification systems appears in published literature in fields other than health. For example, Day and Lloyd describe technology affordances in an educational context [14], Jordan presents a classification scheme based on psychological theory (physical, psychological, social, cognitive) [43], and Zhang explores information-communication technology (ICT) from a motivation perspective [44]. Other attempts within an ICT context have paired user with design, describing affordances as action, functional, and structural [45].

A classification system proposed in [13] may be more relevant to the present study. The resulting schema is that of physical, cognitive, affective, and control affordances. “Physical” are broadly defined as the physical attributes of social media that the user can manipulate (ie, sound and display settings). “Cognitive” are defined as intellectual attributes aiding or facilitating mental processing, or problem solving. They describe the ability to provide information, reinforcement, and suggestions. “Affective” comes from a psychosocial standpoint, related to the attributes of social media that stimulate emotional responses. Finally, “control” exists in social media’s ability to allow the user to make autonomous choices regarding their interactions. For example, one’s level of self-presentation to others will to some extent be dictated by the degree of control allowed by the platform. Control is described as a powerful affordance funneling positive behavioral change, as users who feel they have more control over how they use social media will generally behave more positively [13].

While the present study has appropriated affordance to describe how social media mediate therapeutic effects, the above categories (in particular, cognitive, affective, and control) may still prove useful as a way to theorize the precise mechanisms through which therapeutic affordances work (Table 4). It is also prudent to note that therapeutic affordances mean different things to different people and interpretation may vary. For this reason, those outlined in the present study are a guide and may serve as a base for further investigation.

**Table 4:** Categorization of therapeutic affordances.

	Physical	Cognitive	Affective	Control
Self-presentation				x
Connection			x	x
Exploration		x		x
Narration		x	x	
Adaptation			x	x

“Self-presentation” may be seen as a “control” affordance. Through identification of discourse themes highlighting control, identity, and personal disclosure, as well as language such as anonymity and privacy, it is possible that the self-presentation afforded by social media centers around the ability to “control” online interactions.

Themes identified describing “connection” suggest that much of this affordance comes from the ability to give or receive support and interact with others. Participants spoke of the benefits derived from being able to communicate, discuss, advise, and support each other. This had a beneficial impact on sensations of loneliness and isolation, representing a high socio-emotional component. Thus “affective” may be an appropriate categorization. However, the ability to communicate and generate exchanges with others where and when it suits also describes an element of “control” afforded to users, thus “control” is also a suggested classification.

“Exploration” centered much more on the quest to find information, to find, look, and learn, all in the hope of improving disease-specific knowledge. Hence, exploration is possibly best categorized as a “cognitive” affordance. Concurrently, participants had a strong expectation that the information presented on social media be reputable and that in order to do this, moderation and monitoring of online activity is favored. Hence, “exploration” may also fit in the “control” category.

The “narrative” effect speaks primarily of the emotional catharsis that comes with learning from others’ experiences and sharing one’s own. Hence, suggested categorization as “affective” may be appropriate. However, the strength of the “narration” afforded by social media is also present in how social media allows individuals to develop understanding. This is also true of the personal benefits reported from imparting wisdom to others. For this reason, “narration” may also classify as a “cognitive” affordance.

Finally, the “adaptation” afforded by social media to allow users to evolve their self-management behaviors based on disease-specific needs at different points in time highlights how use changes depending on motivations. For this reason, “affective” is possibly applicable because of the personal or emotional component dictating people’s decision-making process. The majority of participants indicated that social media was particularly useful during times of declining health or pain. Social media gave them greater choice of how they interacted with others. Therefore, categorization as a “control” affordance is also suggested.

### Limitations

Exploring therapeutic affordances has certain limitations. A 2007 paper addressed difficulties with the affordance concept. In that study, a teacher presented her students with a wiki project [14]. The teacher had learned that a primary affordance of wikis is collaboration, which is commonly described in academic literature about wikis [46]. Despite some students embracing the project, the majority did not. While collaboration is a primary affordance of wikis, this example highlights the challenges in realizing the benefits of affordances under different circumstances. Sometimes other factors present within a given context can interfere with the realization or utility of a given affordance. Modeling and categorizing affordances only go part of the way. The present study does not take into account a plethora of contextual factors that can play a role in determining why therapeutic affordances may explain how social media generate health outcomes. For example, one such prominent factor is the characteristics of the group or individual. Day and Lloyd [14] suggest that these may include previous knowledge, competence, learning preferences, motivation, attitudes, and access to information. When placed in the current context (chronic pain management), the same can be applied [14]. However, particular disease-specific characteristics such as current health status, confounding illness variables, and self-management needs also play a vital part.

Also, the platforms used do not function independently of the context in which they are placed [13]. In regards to this research, it is extremely difficult to infer the most suitable social media to meet particular needs and/or objectives in chronic pain management [13,14,47]. Systematic analysis needs to go beyond an elementary view of the social media available for use and the health outcomes expected. It must consider the ways in which the technology (social media) and factors relevant to the individual interplay and contribute to the ensuing interaction and ultimately, outcomes observed. Only then can the gap between potential for use, actual use, and outcomes be bridged.

Social media presents an extra challenge to health research. Unlike traditional clinical health indicators, it is difficult to measure clinical impact of social media on health outcomes reliably. Validating self-reported health outcomes from social media use still requires effort to establish a sound, reputable evidence base. Formal measurement of PROs is required to assess whether social media use based on therapeutic affordances may be effective for improving health outcomes in chronic disease [48]. In order to do this effectively, future research harnessing information about the therapeutic affordances of social media combined with a validated PROs tool is needed to specifically measure health outcomes [49]. The broader chronic disease landscape in particular pushes us to establish valid PROs measurement research methodologies. This research is currently limited in its transferability to other chronic conditions. We have investigated social media use in relation to chronic pain, with chronic pain being chosen due to it burgeoning effect globally, social stigmatization, and co-existence to and manifestation in a variety of chronic conditions [50]. In order to gain a clearer picture of the utility of therapeutic affordances when examining other chronic conditions (eg, cancer, diabetes, arthritis, depression, fibromyalgia), further research is required. Similarly, it is prudent to acknowledge the influence of self-selection on this research [36,51]. To gain a deeper understanding of the influence of social media’s therapeutic affordances, future research may be well served to examine non-responders and perceptions of therapeutic affordances among general social media users in a non-health context [51].

### Conclusions

The results of this paper suggest that future social media research in health may be aided by paying closer attention to therapeutic affordances. By doing so, efficacy may become more discernable. Therapeutic affordances most recognizable in the present study include social media’s ability to “explore” information, “connect” people, and “narrate” experiences of illness. Therapeutic affordances are presented in a model to theorize how the interactions enabled by social media may help explain how health outcomes are generated when individuals use social media as part of health self-management. This approach may provide more targeted, evidence-based recommendations for social media use in chronic disease management. Further research to explore for other therapeutic affordances and study across a range of chronic conditions is warranted to build evidence and move one step closer to producing evidence-based guidelines for social media use in chronic disease management.

## Multimedia Appendix 1

Survey instrument.

## Multimedia Appendix 2

Visualization of full dataset.

## Abbreviations

HCI: human-computer interaction
PROs: patient-reported outcomes
PWCP: people with chronic pain
SNS: social network sites
TCA: thematic content analysis
