# Supplementary material for: Therapeutic Affordances of Social Media: Emergent Themes From a Global Online Survey of People With Chronic Pain
Source: J Med Internet Res. 2014 Dec 22;16(12):e284. doi: 10.2196/jmir.3494 (PMC4285739; doi:10.2196/jmir.3494)
Supplement: Supplementary file 1 [file jmir_v16i12e284_app1.pdf]

<b>

WELCOME - PLAIN LANGUAGE STATEMENT

We would like to invite you to participate in our research project. This **survey** is part of a PhD project from the Health & Biomedical Informatics Centre (the University of Melbourne).

The aim is to better understand use of **social media** for the management of **chronic pain**. The University's Human Research Ethics Committee has approved this project.

***Please note:** Social Media refer to Internet-based platforms that allow for a dynamic Internet experience of communication, sharing & content collaboration (e.g. social network sites, blogs, video sharing, wikis, etc)

You are eligible to participate if:
• You are 18 or older
• Have chronic pain
• Have used social media as part of self-management

What will I be asked to do? (The survey will take approx 15-20 mins). You will be asked about:
• Some personal details
• Your chronic condition & pain
• Your health status
• Your usage of social media for self-management of your condition
• Some specifics about how you use social media for this condition

How will my confidentiality be protected?

The information collected is for research purposes only. We don't want you to give your name, address, other identifiable personal information or health records. The information you supply will be completely anonymous to maintain confidentiality. We intend to protect your anonymity to the fullest possible extent within the limits of the law. However, please note that while we do our utmost to do this, it is possible for data to be subject to subpoena, freedom of information request or mandated reporting by some professions.

The only people with access to the information you supply in the survey will be the research team and all data will be stored securely under password protection online and securely within our research unit. It will be stored for five years from the date of results publication, before being destroyed.

How will I receive feedback?

Once the research findings are published in an academic journal, the link to the article will be available via our website and social media channels at the Health and Biomedical Informatics Centre. Please visit our website for details of our social media platforms (http://www.healthinformatics.unimelb.edu.au/)

Do I have to take part?

Participation is voluntary. Nobody affiliated to your support organization or group will be notified or have any awareness to your participation. Also, you can choose to stop the survey and withdraw

after commencing, but withdrawal will have to occur before finishing and submitting the survey.

***Please note:**
- We only ask for anonymous information. Please don't tell us anything that will identify you
- This survey will require approx. 15-20 mins of computer use. We suggest that if you don't feel able to do this or are concerned participating may exacerbate your condition, please don't participate
- This is a survey only and doesn't provide or substitute professional medical advice, nor suggest usage practices of social media. Please contact your support group, organization and/or health professional for more advice.

Where can I get further information?

Should you require further information, or have any concerns, please contact the researchers on +613 9035 4943. Should you have any concerns about the conduct of the project, you are welcome to contact the Manager, Human Research Ethics, University of Melbourne (ph: +613 8344 2073 fax: +613 9347 6739)

How do I agree to participate?

If you would like to participate, **view the accompanying consent form on the next page**. By clicking through from the consent form to the survey, you are indicating you have read and understood this information and agree to participate.

<b>

INFORMED CONSENT FORM

<u>Name of investigator(s): Dr. Kathleen Gray, Mr. Mark Merolli, Prof Fernando Martin-Sanchez</u>

1. I consent to participate in this project, the details of which have been explained to me, and I have viewed the plain language statement, which I can print if I require.

2. I understand that by completing the accompanying survey, I am giving consent

3. By consenting, I am indicating that I am at least 18 years of age, suffer chronic pain and have used social media as part of self-management

4. I understand that my participation will involve a survey and I agree that the researcher may use the results for the purposes described in the plain language statement.

5. I acknowledge that:

(a) the possible effects of participating in the survey have been explained to my satisfaction;

(b) I have been informed that I am free to withdraw from the survey before submitting without explanation or prejudice

(c) the project is for the purpose of research into social media use in chronic disease;

(d) I have been informed that the information I provide will be entirely anonymous data to maintain confidentiality and safeguarded subject to any legal requirements;

(e) The survey will contain no identifiable personal information about me and/or my health status

(f) I have been informed that with my consent the survey will be retained at the University of Melbourne and will be destroyed after five years;

(g) I have been informed that a copy of the research findings may be published in an academic journal or presented at academic conferences. If publication occurs, it will be made known via the Health and Biomedical informatics Centre’s (the University of Melbourne) website

***By clicking 'next' from this form to the questions and submitting the survey you are indicating that you have read and understood this information and the information contained in the Plain Language Statement, and thus agree to participate in this study.**

<b>

DEMOGRAPHIC INFORMATION

Please read the instructions carefully before answering the questions.

Thank you for your time

1. What is your gender?

- ◯ Female
- ◯ Male

2. Please tell us your age range (choose what is most appropriate):

- ◯ 18-19
- ◯ 20-29
- ◯ 30-39
- ◯ 40-49
- ◯ 50-59
- ◯ 60-69
- ◯ 70+

3. What is your current marital status?

- ◯ Never married
- ◯ Currently married
- ◯ Partnered
- ◯ Separated
- ◯ Divorced
- ◯ Widowed

4. What is the highest level of education that you have completed?

- ◯ No formal schooling
- ◯ Less than primary/elementary school
- ◯ Primary/elementary school completed
- ◯ High school (or equivalent) completed
- ◯ College/university completed
- ◯ Post-graduate degree completed

5. In which country do you live?

	Country:
Please select:	

<b>

6. Are you currently working?

- ◯ Full-time
- ◯ Part-time
- ◯ Not working for pay

<b>

7. If not working for pay, what is the main reason?

- Home maker/caring for family
- Looked but can’t find a job
- Doing unpaid work/voluntary activities
- Studies/training
- Retired/too old to work
- ill health

Other (please specify)

8. Where did you hear about this survey?

Please select:

Other (please specify):

<b>

INFORMATION ABOUT YOUR CONDITION

We would like to ask you some questions about your chronic pain and/or other medical conditions

***9. Do you have chronic pain (pain over 3 months duration)?**

◯ Yes

◯ No

10. Has this been formally diagnosed?

◯ Yes

◯ No

◯ Don't Know

11. If you have you been undergoing treatment for your chronic pain during the last year, please select those that apply to you, otherwise leave blank (you can select more than one):

☐ Medication

☐ Doctor visits

☐ Pain Clinic

☐ Physical Therapy/Physiotherapy

☐ Exercise classes

☐ Psychology/counseling

☐ Relaxation/meditation

Other (please specify)

12. Have you been formally diagnosed with a chronic disease that has led to your pain?

◯ Yes

◯ No

◯ Don't Know

<b>

13. If 'yes', what is the condition?

	Condition:
Please select:	

Other (please specify)

<b>

CHRONIC PAIN

The following questions relate specifically to your chronic pain:

Please respond to each item by marking one box per row

IN THE PAST SEVEN DAYS....

14. How would you rate your pain on average?

	0 (no pain)	1	2	3	4	5	6	7	8	9	10 (worst imaginable pain)
Please select:	◯	◯	◯	◯	◯	◯	◯	◯	◯	◯	◯

15. How difficult was it for you to take in new information because of pain?

	Not at all	A little bit	Somewhat	Quite a bit	Very much
Please select:	◯	◯	◯	◯	◯

16. How much did pain interfere with your ability to concentrate?

	Not at all	A little bit	Somewhat	Quite a bit	Very much
Please select:	◯	◯	◯	◯	◯

17. How much did pain make it difficult to fall asleep?

	Not at all	A little bit	Somewhat	Quite a bit	Very much
Please select:	◯	◯	◯	◯	◯

18. How much did pain interfere with your enjoyment of life?

	Not at all	A little bit	Somewhat	Quite a bit	Very much
Please select:	◯	◯	◯	◯	◯

19. How much did pain interfere with your ability to participate in social activities?

	Not at all	A little bit	Somewhat	Quite a bit	Very much
Please select:	◯	◯	◯	◯	◯

20. How much did pain interfere with your relationships with other people?

	Not at all	A little bit	Somewhat	Quite a bit	Very much
Please select:	◯	◯	◯	◯	◯

21. How much did pain interfere with your family life?

	Not at all	A little bit	Somewhat	Quite a bit	Very much
Please select:	◯	◯	◯	◯	◯

22. How much did pain interfere with your day to day activities?

	Not at all	A little bit	Somewhat	Quite a bit	Very much
Please select:	◯	◯	◯	◯	◯

<b>

23. How much did pain interfere with your household chores?

	Not at all	A little bit	Somewhat	Quite a bit	Very much
Please select:	○	○	○	○	○

24. How much did pain interfere with your ability to work (include work at home)?

	Not at all	A little bit	Somewhat	Quite a bit	Very much
Please select:	○	○	○	○	○

25. How much did pain feel like a burden to you?

	Not at all	A little bit	Somewhat	Quite a bit	Very much
Please select:	○	○	○	○	○

26. How often did pain make you feel anxious?

	Never	Rarely	Sometimes	Often	Always
Please select:	○	○	○	○	○

27. How often did pain make you feel depressed?

	Never	Rarely	Sometimes	Often	Always
Please select:	○	○	○	○	○

28. How often did pain prevent you from sitting for more than 30 minutes?

	Never	Rarely	Sometimes	Often	Always
Please select:	○	○	○	○	○

29. How often did pain prevent you from standing for more than 30 minutes?

	Never	Rarely	Sometimes	Often	Always
Please select:	○	○	○	○	○

30. How often did pain prevent you from walking more than 1 mile (1.6 km)?

	Never	Rarely	Sometimes	Often	Always
Please select:	○	○	○	○	○

<b>

SOCIAL MEDIA USE

In the following section you will only be asked to provide detail for **THOSE PLATFORMS YOU HAVE USED** as part of your chronic pain self-management

If the platform is not applicable to you, select **NO**

<b>

SOCIAL NETWORK SITES

The following questions relate specifically to **SOCIAL NETWORK SITES**

Description: Social Network Sites are online community platforms that allow users to connect and share interests and/or activities

Examples include but are not limited to: Facebook, Mypace, Patients Like Me, Daily Strength, etc

***31. In the last year, have you used SOCIAL NETWORK SITES when you go online for information, communication or interaction about your chronic pain?**

- ◯ Yes
- ◯ No

32. How valuable are SOCIAL NETWORK SITES for information, communication or interaction about your chronic pain?

◯ Not at all ◯ A little bit ◯ Somewhat ◯ Quite a bit ◯ Very much

33. How often do you use SOCIAL NETWORK SITES as part of your chronic pain self-management?

	At least daily	At least weekly	At least monthly	Less than once a month
Please select:	◯	◯	◯	◯

34. Please select the specific things you do on SOCIAL NETWORK SITES for your chronic pain self-management (you can select more than one):

- ☐ Status update
- ☐ Post to someone's profile/page
- ☐ Like a comment or post
- ☐ Comment on someone's post/content
- ☐ Tag content
- ☐ Private message someone
- ☐ Add a friend/connection
- ☐ Share content
- ☐ Chat

Other (please specify)

____

With your use of SOCIAL NETWORK SITES for chronic pain self-management in mind, please indicate your level of agreement with the following statements..

Note: If you feel a statement is **'NOT APPLICABLE'** to this platform, **LEAVE IT BLANK**

35. I prefer to control the amount and sorts of things that other people know about me on SOCIAL NETWORK SITES

	Strongly Agree	Agree	Neither agree or disagree	Disagree	Strongly Disagree
Please select:	◯	◯	◯	◯	◯

<b>

36. I prefer to know personal information about the people I interact with on SOCIAL NETWORK SITES

	Strongly Agree	Agree	Neither agree or disagree	Disagree	Strongly Disagree
Please select:	◯	◯	◯	◯	◯

37. I prefer to remain anonymous on SOCIAL NETWORK SITES

	Strongly Agree	Agree	Neither agree or disagree	Disagree	Strongly Disagree
Please select:	◯	◯	◯	◯	◯

38. I value the ability to do the activities I want on SOCIAL NETWORK SITES at any time I choose/when it suits me

	Strongly Agree	Agree	Neither agree or disagree	Disagree	Strongly Disagree
Please select:	◯	◯	◯	◯	◯

39. I prefer to use SOCIAL NETWORK SITES only when the people I interact with are online at the exact same time

	Strongly Agree	Agree	Neither agree or disagree	Disagree	Strongly disagree
Please select:	◯	◯	◯	◯	◯

40. I value the ability to use SOCIAL NETWORK SITES not just at home but when I am elsewhere too

	Strongly Agree	Agree	Neither agree or disagree	Disagree	Strongly Disagree
Please select:	◯	◯	◯	◯	◯

41. I value the way SOCIAL NETWORK SITES act as a kind of filter to guide me to useful information

	Strongly Agree	Agree	Neither agree or disagree	Disagree	Strongly Disagree
Please select:	◯	◯	◯	◯	◯

42. I value it when there is a health professional contributing to SOCIAL NETWORK SITE use

	Strongly Agree	Agree	Neither agree or disagree	Disagree	Strongly Disagree
Please select:	◯	◯	◯	◯	◯

43. I value it when there is a group facilitator/moderator overseeing SOCIAL NETWORK SITE use

	Strongly Agree	Agree	Neither agree or disagree	Disagree	Strongly Disagree
Please select:	◯	◯	◯	◯	◯

<b>

44. SOCIAL NETWORK SITES are an effective platform to record stories of chronic pain (yours and other people's)

	Strongly Agree	Agree	Neither agree or disagree	Disagree	Strongly Disagree
Please select:	◯	◯	◯	◯	◯

45. I value being able to share my experiences through SOCIAL NETWORK SITES

	Strongly Agree	Agree	Neither agree or disagree	Disagree	Strongly Disagree
Please select:	◯	◯	◯	◯	◯

46. I value being able to find out about others' experiences through SOCIAL NETWORK SITES

	Strongly Agree	Agree	Neither agree or disagree	Disagree	Strongly Disagree
Please select:	◯	◯	◯	◯	◯

47. I use SOCIAL NETWORK SITES differently depending on the stage of my condition

	Strongly Agree	Agree	Neither agree or disagree	Disagree	Strongly Disagree
Please select:	◯	◯	◯	◯	◯

48. When my condition is severe or flared-up, I use SOCIAL NETWORK SITES..

	Never	Almost never	Sometimes	Fairly often	Very often
Please select:	◯	◯	◯	◯	◯

49. When my condition is stable, I use SOCIAL NETWORK SITES..

	Never	Almost never	Sometimes	Fairly often	Very often
Please select:	◯	◯	◯	◯	◯

<b>

50. Do you feel that your use of SOCIAL NETWORK SITES has in any way helped your..

<u>Note:</u> You only need to respond to those items relevant to your situation

	Very much	Quite a bit	Somewhat	A little bit	Not at all
Ability to take in new information	○	○	○	○	○
Ability to concentrate	○	○	○	○	○
Ability to sleep	○	○	○	○	○
Enjoyment of life	○	○	○	○	○
Ability to participate in social activities	○	○	○	○	○
Relationships with other people	○	○	○	○	○
Family life	○	○	○	○	○
Ability to perform your day-to-day activities	○	○	○	○	○
Ability to do household chores	○	○	○	○	○
Ability to work (include work at home)	○	○	○	○	○
Emotional burden	○	○	○	○	○
Anxiety	○	○	○	○	○
Depression	○	○	○	○	○
Ability to sit for more than 30 minutes	○	○	○	○	○
Ability to stand for more than 30 minutes	○	○	○	○	○
Ability to walk more than 1 mile (1.6 km)	○	○	○	○	○

51. Please use this space if you would like to comment further about your use of SOCIAL NETWORK SITES for your chronic pain self-management..

<b>

BLOGS

The following questions relate specifically to **BLOGS**

Description: Blogs are platforms that allow users to post information, narratives or generate discussion

Examples include but are not limited to: Blogger, Wordpress, etc

***52. In the last year, have you used BLOGS when you go online for information, communication or interaction about your chronic pain?**

- ◯ Yes
- ◯ No

<b>

53. How valuable are BLOGS for information, communication or interaction about your chronic pain?

◯ Not at all ◯ A little bit ◯ Somewhat ◯ Quite a bit ◯ Very much

54. How often do you use BLOGS as part of your chronic pain self-management?

	At least daily	At least weekly	At least monthly	Less than once a month
Please select:	◯	◯	◯	◯

55. Please select the specific things you do on BLOGS for your chronic pain self-management? (you can select more than one):

- ☐ Read a blog
- ☐ Post my own blog
- ☐ Comment on a blog post
- ☐ Share a blog post
- ☐ Subscribe to a blog

Other (please specify)

With your use of BLOGS for chronic pain self-management in mind, please indicate your level of agreement with the following statements..

Note: If you feel a statement is **'NOT APPLICABLE'** to this platform, **LEAVE IT BLANK**

56. I prefer to control the amount and sorts of things that other people know about me on BLOGS

	Strongly Agree	Agree	Neither agree or disagree	Disagree	Strongly Disagree
Please select:	◯	◯	◯	◯	◯

57. I prefer to know personal information about the people I interact with on BLOGS

	Strongly Agree	Agree	Neither agree or disagree	Disagree	Strongly Disagree
Please select:	◯	◯	◯	◯	◯

58. I prefer to remain anonymous on BLOGS

	Strongly Agree	Agree	Neither agree or disagree	Disagree	Strongly Disagree
Please select:	◯	◯	◯	◯	◯

<b>

59. I value the ability to do the activities I want on BLOGS at any time I choose/when it suits me

	Strongly Agree	Agree	Neither agree or disagree	Disagree	Strongly Disagree
Please select:	◯	◯	◯	◯	◯

60. I prefer to use BLOGS only when the people I interact with are online at the exact same time

	Strongly Agree	Agree	Neither agree or disagree	Disagree	Strongly disagree
Please select:	◯	◯	◯	◯	◯

61. I value the ability to use BLOGS not just at home but when I am elsewhere too

	Strongly Agree	Agree	Neither agree or disagree	Disagree	Strongly Disagree
Please select:	◯	◯	◯	◯	◯

62. I value the way BLOGS act as a kind of filter to guide me to useful information

	Strongly Agree	Agree	Neither agree or disagree	Disagree	Strongly Disagree
Please select:	◯	◯	◯	◯	◯

63. I value it when there is a health professional contributing to BLOG use

	Strongly Agree	Agree	Neither agree or disagree	Disagree	Strongly Disagree
Please select:	◯	◯	◯	◯	◯

64. I value it when there is a group facilitator/moderator overseeing BLOG use

	Strongly Agree	Agree	Neither agree or disagree	Disagree	Strongly Disagree
Please select:	◯	◯	◯	◯	◯

65. BLOGS are an effective platform to record stories of chronic pain (yours and other people's)

	Strongly Agree	Agree	Neither agree or disagree	Disagree	Strongly Disagree
Please select:	◯	◯	◯	◯	◯

66. I value being able to share my experiences through BLOGS

	Strongly Agree	Agree	Neither agree or disagree	Disagree	Strongly Disagree
Please select:	◯	◯	◯	◯	◯

67. I value being able to find out about others' experiences through BLOGS

	Strongly Agree	Agree	Neither agree or disagree	Disagree	Strongly Disagree
Please select:	◯	◯	◯	◯	◯

<b>

68. I use BLOGS differently depending on the stage of my condition

	Strongly Agree	Agree	Neither agree or disagree	Disagree	Strongly Disagree
Please select:	◯	◯	◯	◯	◯

69. When my condition is severe or flared-up, I use BLOGS..

	Never	Almost never	Sometimes	Fairly often	Very often
Please select:	◯	◯	◯	◯	◯

70. When my condition is stable, I use BLOGS..

	Never	Almost never	Sometimes	Fairly often	Very often
Please select:	◯	◯	◯	◯	◯

<b>

71. Do you feel that your use of BLOGS has in any way helped your..

<u>Note:</u> You only need to respond to those items relevant to your situation

	Very much	Quite a bit	Somewhat	A little bit	Not at all
Ability to take in new information	◯	◯	◯	◯	◯
Ability to concentrate	◯	◯	◯	◯	◯
Ability to sleep	◯	◯	◯	◯	◯
Enjoyment of life	◯	◯	◯	◯	◯
Ability to participate in social activities	◯	◯	◯	◯	◯
Relationships with other people	◯	◯	◯	◯	◯
Family life	◯	◯	◯	◯	◯
Ability to perform your day-to-day activities	◯	◯	◯	◯	◯
Ability to do household chores	◯	◯	◯	◯	◯
Ability to work (include work at home)	◯	◯	◯	◯	◯
Emotional burden	◯	◯	◯	◯	◯
Anxiety	◯	◯	◯	◯	◯
Depression	◯	◯	◯	◯	◯
Ability to sit for more than 30 minutes	◯	◯	◯	◯	◯
Ability to stand for more than 30 minutes	◯	◯	◯	◯	◯
Ability to walk more than 1 mile (1.6 km)	◯	◯	◯	◯	◯

72. Please use this space if you would like to comment further about your use of BLOGS for your chronic pain self-management..

<b>

WIKIS

The following questions relate specifically to **WIKIS**

Description: Wikis are shared sites/platforms of collections of linked webpages that enable users to collaborate and coordinate information

Examples include but are not limited to: Wikipedia, Wikia, etc

***73. In the last year, have you used WIKIS when you go online for information, communication or interaction about your chronic pain?**

◯ Yes

◯ No

<b>

74. How valuable are WIKIS for information, communication or interaction about your chronic pain?

○ Not at all ○ A little bit ○ Somewhat ○ Quite a bit ○ Very much

75. How often do you use WIKIS as part of your chronic pain self-management?

	At least daily	At least weekly	At least monthly	Less than once a month
Please select:	○	○	○	○

76. Please select the specific things you do on WIKIS for your chronic pain self-management? (you can select more than one):

- ☐ Read a wiki
- ☐ Start a wiki
- ☐ Contribute to a wiki
- ☐ Edit a wiki
- ☐ Share a wiki

Other (please specify)

With your use of WIKIS for chronic pain self-management in mind, please indicate your level of agreement with the following statements..

Note: If you feel a statement is **'NOT APPLICABLE'** to this platform, **LEAVE IT BLANK**

77. I prefer to control the amount and sorts of things that other people know about me on WIKIS

	Strongly Agree	Agree	Neither agree or disagree	Disagree	Strongly Disagree
Please select:	○	○	○	○	○

78. I prefer to know personal information about the people I interact with on WIKIS

	Strongly Agree	Agree	Neither agree or disagree	Disagree	Strongly Disagree
Please select:	○	○	○	○	○

79. I prefer to remain anonymous on WIKIS

	Strongly Agree	Agree	Neither agree or disagree	Disagree	Strongly Disagree
Please select:	○	○	○	○	○

<b>

80. I value the ability to do the activities I want on WIKIS at any time I choose/when it suits me

	Strongly Agree	Agree	Neither agree or disagree	Disagree	Strongly Disagree
Please select:	◯	◯	◯	◯	◯

81. I prefer to use WIKIS only when the people I interact with are online at the exact same time

	Strongly Agree	Agree	Neither agree or disagree	Disagree	Strongly disagree
Please select:	◯	◯	◯	◯	◯

82. I value the ability to use WIKIS not just at home but when I am elsewhere too

	Strongly Agree	Agree	Neither agree or disagree	Disagree	Strongly Disagree
Please select:	◯	◯	◯	◯	◯

83. I value the way WIKIS act as a kind of filter to guide me to useful information

	Strongly Agree	Agree	Neither agree or disagree	Disagree	Strongly Disagree
Please select:	◯	◯	◯	◯	◯

84. I value it when there is a health professional contributing to WIKI use

	Strongly Agree	Agree	Neither agree or disagree	Disagree	Strongly Disagree
Please select:	◯	◯	◯	◯	◯

85. I value it when there is a group facilitator/moderator overseeing WIKI use

	Strongly Agree	Agree	Neither agree or disagree	Disagree	Strongly Disagree
Please select:	◯	◯	◯	◯	◯

86. WIKIS are an effective platform to record stories of chronic pain (yours and other people's)

	Strongly Agree	Agree	Neither agree or disagree	Disagree	Strongly Disagree
Please select:	◯	◯	◯	◯	◯

87. I value being able to share my experiences through WIKIS

	Strongly Agree	Agree	Neither agree or disagree	Disagree	Strongly Disagree
Please select:	◯	◯	◯	◯	◯

88. I value being able to find out about others' experiences through WIKIS

	Strongly Agree	Agree	Neither agree or disagree	Disagree	Strongly Disagree
Please select:	◯	◯	◯	◯	◯

<b>

89. I use WIKIS differently depending on the stage of my condition

	Strongly Agree	Agree	Neither agree or disagree	Disagree	Strongly Disagree
Please select:	◯	◯	◯	◯	◯

90. When my condition is severe or flared-up, I use WIKIS..

	Never	Almost never	Sometimes	Fairly often	Very often
Please select:	◯	◯	◯	◯	◯

91. When my condition is stable, I use WIKIS..

	Never	Almost never	Sometimes	Fairly often	Very often
Please select:	◯	◯	◯	◯	◯

<b>

92. Do you feel that your use of WIKIS has in any way helped your..

<u>Note:</u> You only need to respond to those items relevant to your situation

	Very much	Quite a bit	Somewhat	A little bit	Not at all
Ability to take in new information	◯	◯	◯	◯	◯
Ability to concentrate	◯	◯	◯	◯	◯
Ability to sleep	◯	◯	◯	◯	◯
Enjoyment of life	◯	◯	◯	◯	◯
Ability to participate in social activities	◯	◯	◯	◯	◯
Relationships with other people	◯	◯	◯	◯	◯
Family life	◯	◯	◯	◯	◯
Ability to perform your day-to-day activities	◯	◯	◯	◯	◯
Ability to do household chores	◯	◯	◯	◯	◯
Ability to work (include work at home)	◯	◯	◯	◯	◯
Emotional burden	◯	◯	◯	◯	◯
Anxiety	◯	◯	◯	◯	◯
Depression	◯	◯	◯	◯	◯
Ability to sit for more than 30 minutes	◯	◯	◯	◯	◯
Ability to stand for more than 30 minutes	◯	◯	◯	◯	◯
Ability to walk more than 1 mile (1.6 km)	◯	◯	◯	◯	◯

93. Please use this space if you would like to comment further about your use of WIKIS for your chronic pain self-management..

<b>

MICROBLOGS

The following questions relate specifically to **MICROBLOGS**

Description: Microblogs are a short form of blogging that allow users to send brief information, short status updates or media to be viewed publicly or privately

Examples include but are not limited to: Twitter, Tumblr, etc

***94. In the last year, have you used MICROBLOGS when you go online for information, communication or interaction about your chronic pain?**

- ◯ Yes
- ◯ No

<b>

95. How valuable are MICROBLOGS for information, communication or interaction about your chronic pain?

◯ Not at all ◯ A little bit ◯ Somewhat ◯ Quite a bit ◯ Very much

96. How often do you use MICROBLOGS as part of your chronic pain self-management?

	At least daily	At least weekly	At least monthly	Less than once a month
Please select:	◯	◯	◯	◯

97. Please select the specific things you do on MICROBLOGS for your chronic pain self-management? (you can select more than one):

- ☐ Follow another user
- ☐ Direct message other users
- ☐ Read posts by others
- ☐ Post an update or media yourself

Other (please specify)

With your use of MICROBLOGS for chronic pain self-management in mind, please indicate your level of agreement with the following statements..

Note: If you feel a statement is **'NOT APPLICABLE'** to this platform, **LEAVE IT BLANK**

98. I prefer to control the amount and sorts of things that other people know about me on MICROBLOGS

	Strongly Agree	Agree	Neither agree or disagree	Disagree	Strongly Disagree
Please select:	◯	◯	◯	◯	◯

99. I prefer to know personal information about the people I interact with on MICROBLOGS

	Strongly Agree	Agree	Neither agree or disagree	Disagree	Strongly Disagree
Please select:	◯	◯	◯	◯	◯

100. I prefer to remain anonymous on MICROBLOGS

	Strongly Agree	Agree	Neither agree or disagree	Disagree	Strongly Disagree
Please select:	◯	◯	◯	◯	◯

<b>

101. I value the ability to do the activities I want on MICROBLOGS at any time I choose/when it suits me

	Strongly Agree	Agree	Neither agree or disagree	Disagree	Strongly Disagree
Please select:	○	○	○	○	○

102. I prefer to use MICROBLOGS only when the people I interact with are online at the exact same time

	Strongly Agree	Agree	Neither agree or disagree	Disagree	Strongly disagree
Please select:	○	○	○	○	○

103. I value the ability to use MICROBLOGS not just at home but when I am elsewhere too

	Strongly Agree	Agree	Neither agree or disagree	Disagree	Strongly Disagree
Please select:	○	○	○	○	○

104. I value the way MICROBLOGS act as a kind of filter to guide me to useful information

	Strongly Agree	Agree	Neither agree or disagree	Disagree	Strongly Disagree
Please select:	○	○	○	○	○

105. I value it when there is a health professional contributing to MICROBLOG use

	Strongly Agree	Agree	Neither agree or disagree	Disagree	Strongly Disagree
Please select:	○	○	○	○	○

106. I value it when there is a group facilitator/moderator overseeing MICROBLOG use

	Strongly Agree	Agree	Neither agree or disagree	Disagree	Strongly Disagree
Please select:	○	○	○	○	○

107. MICROBLOGS are an effective platform to record stories of chronic pain (yours and other people's)

	Strongly Agree	Agree	Neither agree or disagree	Disagree	Strongly Disagree
Please select:	○	○	○	○	○

108. I value being able to share my experiences through MICROBLOGS

	Strongly Agree	Agree	Neither agree or disagree	Disagree	Strongly Disagree
Please select:	○	○	○	○	○

<b>

109. I value being able to find out about others' experiences through MICROBLOGS

	Strongly Agree	Agree	Neither agree or disagree	Disagree	Strongly Disagree
Please select:	◯	◯	◯	◯	◯

110. I use MICROBLOGS differently depending on the stage of my condition

	Strongly Agree	Agree	Neither agree or disagree	Disagree	Strongly Disagree
Please select:	◯	◯	◯	◯	◯

111. When my condition is severe or flared-up, I use MICROBLOGS..

	Never	Almost never	Sometimes	Fairly often	Very often
Please select:	◯	◯	◯	◯	◯

112. When my condition is stable, I use MICROBLOGS..

	Never	Almost never	Sometimes	Fairly often	Very often
Please select:	◯	◯	◯	◯	◯

<b>

113. Do you feel that your use of MICROBLOGS has in any way helped your..

Note: You only need to respond to those items relevant to your situation

	Very much	Quite a bit	Somewhat	A little bit	Not at all
Ability to take in new information	◯	◯	◯	◯	◯
Ability to concentrate	◯	◯	◯	◯	◯
Ability to sleep	◯	◯	◯	◯	◯
Enjoyment of life	◯	◯	◯	◯	◯
Ability to participate in social activities	◯	◯	◯	◯	◯
Relationships with other people	◯	◯	◯	◯	◯
Family life	◯	◯	◯	◯	◯
Ability to perform your day-to-day activities	◯	◯	◯	◯	◯
Ability to do household chores	◯	◯	◯	◯	◯
Ability to work (include work at home)	◯	◯	◯	◯	◯
Emotional burden	◯	◯	◯	◯	◯
Anxiety	◯	◯	◯	◯	◯
Depression	◯	◯	◯	◯	◯
Ability to sit for more than 30 minutes	◯	◯	◯	◯	◯
Ability to stand for more than 30 minutes	◯	◯	◯	◯	◯
Ability to walk more than 1 mile (1.6 km)	◯	◯	◯	◯	◯

114. Please use this space if you would like to comment further about your use of MICROBLOGS for your chronic pain self-management..

<b>

TAGGING/AGGREGATION SITES

The following questions relate specifically to **TAGGING/AGGREGATION SITES**

Description: Tagging/aggregation sites allow users to save, collect, tag, manage and share links to web content (often in a centralized location). They sometimes also allow users to vote/rate content, resulting in the most popular content being more visible on the site

Examples include but are not limited to: Delicious, Stumble Upon, Digg, Reddit, etc

***115. In the last year, have you used TAGGING/AGGREGATION SITES when you go online for information, communication or interaction about your chronic pain?**

- ◯ Yes
- ◯ No

<b>

116. How valuable are TAGGING/AGGREGATION SITES for information, communication or interaction about your chronic pain?

◯ Not at all ◯ A little bit ◯ Somewhat ◯ Quite a bit ◯ Very much

117. How often do you use TAGGING/AGGREGATION SITES as part of your chronic pain self-management?

	At least daily	At least weekly	At least monthly	Less than once a month
Please select:	◯	◯	◯	◯

118. Please select the specific things you do on TAGGING/AGGREGATION SITES for your chronic pain self-management? (you can select more than one):

- ☐ Tag/bookmark content
- ☐ Rate content
- ☐ Review content
- ☐ Share content

Other (please specify)

____

With your use of TAGGING/AGGREGATION SITES for chronic pain self-management in mind, please indicate your level of agreement with the following statements..

Note: If you feel a statement is **'NOT APPLICABLE'** to this platform, **LEAVE IT BLANK**

119. I prefer to control the amount and sorts of things that other people know about me on TAGGING/AGGREGATION SITES

	Strongly Agree	Agree	Neither agree or disagree	Disagree	Strongly Disagree
Please select:	◯	◯	◯	◯	◯

120. I prefer to know personal information about the people I interact with on TAGGING/AGGREGATION SITES

	Strongly Agree	Agree	Neither agree or disagree	Disagree	Strongly Disagree
Please select:	◯	◯	◯	◯	◯

121. I prefer to remain anonymous on TAGGING/AGGREGATION SITES

	Strongly Agree	Agree	Neither agree or disagree	Disagree	Strongly Disagree
Please select:	◯	◯	◯	◯	◯

<b>

122. I value the ability to do the activities I want on TAGGING/AGGREGATION SITES at any time I choose/when it suits me

	Strongly Agree	Agree	Neither agree or disagree	Disagree	Strongly Disagree
Please select:	◯	◯	◯	◯	◯

123. I prefer to use TAGGING/AGGREGATION SITES only when the people I interact with are online at the exact same time

	Strongly Agree	Agree	Neither agree or disagree	Disagree	Strongly disagree
Please select:	◯	◯	◯	◯	◯

124. I value the ability to use TAGGING/AGGREGATION SITES not just at home but when I am elsewhere too

	Strongly Agree	Agree	Neither agree or disagree	Disagree	Strongly Disagree
Please select:	◯	◯	◯	◯	◯

125. I value the way TAGGING/AGGREGATION SITES act as a kind of filter to guide me to useful information

	Strongly Agree	Agree	Neither agree or disagree	Disagree	Strongly Disagree
Please select:	◯	◯	◯	◯	◯

126. I value it when there is a health professional contributing to TAGGING/AGGREGATION SITE use

	Strongly Agree	Agree	Neither agree or disagree	Disagree	Strongly Disagree
Please select:	◯	◯	◯	◯	◯

127. I value it when there is a group facilitator/moderator overseeing TAGGING/AGGREGATION SITE use

	Strongly Agree	Agree	Neither agree or disagree	Disagree	Strongly Disagree
Please select:	◯	◯	◯	◯	◯

128. TAGGING/AGGREGATION SITES are an effective platform to record stories of chronic pain (yours and other people's)

	Strongly Agree	Agree	Neither agree or disagree	Disagree	Strongly Disagree
Please select:	◯	◯	◯	◯	◯

<b>

129. I value being able to share my experiences through TAGGING/AGGREGATION SITES

	Strongly Agree	Agree	Neither agree or disagree	Disagree	Strongly Disagree
Please select:	◯	◯	◯	◯	◯

130. I value being able to find out about others' experiences through TAGGING/AGGREGATION SITES

	Strongly Agree	Agree	Neither agree or disagree	Disagree	Strongly Disagree
Please select:	◯	◯	◯	◯	◯

131. I use TAGGING/AGGREGATION SITES differently depending on the stage of my condition

	Strongly Agree	Agree	Neither agree or disagree	Disagree	Strongly Disagree
Please select:	◯	◯	◯	◯	◯

132. When my condition is severe or flared-up, I use TAGGING/AGGREGATION SITES..

	Never	Almost never	Sometimes	Fairly often	Very often
Please select:	◯	◯	◯	◯	◯

133. When my condition is stable, I use TAGGING/AGGREGATION SITES..

	Never	Almost never	Sometimes	Fairly often	Very often
Please select:	◯	◯	◯	◯	◯

<b>

134. Do you feel that your use of TAGGING/AGGREGATION SITES has in any way helped your..

<u>Note:</u> You only need to respond to those items relevant to your situation

	Very much	Quite a bit	Somewhat	A little bit	Not at all
Ability to take in new information	◯	◯	◯	◯	◯
Ability to concentrate	◯	◯	◯	◯	◯
Ability to sleep	◯	◯	◯	◯	◯
Enjoyment of life	◯	◯	◯	◯	◯
Ability to participate in social activities	◯	◯	◯	◯	◯
Relationships with other people	◯	◯	◯	◯	◯
Family life	◯	◯	◯	◯	◯
Ability to perform your day-to-day activities	◯	◯	◯	◯	◯
Ability to do household chores	◯	◯	◯	◯	◯
Ability to work (include work at home)	◯	◯	◯	◯	◯
Emotional burden	◯	◯	◯	◯	◯
Anxiety	◯	◯	◯	◯	◯
Depression	◯	◯	◯	◯	◯
Ability to sit for more than 30 minutes	◯	◯	◯	◯	◯
Ability to stand for more than 30 minutes	◯	◯	◯	◯	◯
Ability to walk more than 1 mile (1.6 km)	◯	◯	◯	◯	◯

135. Please use this space if you would like to comment further about your use of TAGGING/AGGREGATION SITES for your chronic pain self-management..

<b>

VIRTUAL ENVIRONMENTS

The following questions relate specifically to **VIRTUAL ENVIRONMENTS**

Description: Virtual Environments are simulated environments that allow users to interact with each other and the surrounding environment

Examples include but are not limited to: Second Life, etc

***136. In the last year, have you used VIRTUAL ENVIRONMENTS when you go online for information, communication or interaction about your chronic pain?**

- ◯ Yes
- ◯ No

<b>

137. How valuable are VIRTUAL ENVIRONMENTS for information, communication or interaction about your chronic pain?

◯ Not at all ◯ A little bit ◯ Somewhat ◯ Quite a bit ◯ Very much

138. How often do you use VIRTUAL ENVIRONMENTS as part of your chronic pain self-management?

	At least daily	At least weekly	At least monthly	Less than once a month
Please select:	◯	◯	◯	◯

139. Please select the specific things you do in VIRTUAL ENVIRONMENTS for your chronic pain self-management? (you can select more than one):

- ☐ Be present but not interact
- ☐ Interact with others
- ☐ Build environments
- ☐ Edit environments

Other (please specify)

With your use of VIRTUAL ENVIRONMENTS for chronic pain self-management in mind, please indicate your level of agreement with the following statements..

Note: If you feel a statement is **'NOT APPLICABLE'** to this platform, **LEAVE IT BLANK**

140. I prefer to control the amount and sorts of things that other people know about me in VIRTUAL ENVIRONMENTS

	Strongly Agree	Agree	Neither agree or disagree	Disagree	Strongly Disagree
Please select:	◯	◯	◯	◯	◯

141. I prefer to know personal information about the people I interact with in VIRTUAL ENVIRONMENTS

	Strongly Agree	Agree	Neither agree or disagree	Disagree	Strongly Disagree
Please select:	◯	◯	◯	◯	◯

142. I prefer to remain anonymous in VIRTUAL ENVIRONMENTS

	Strongly Agree	Agree	Neither agree or disagree	Disagree	Strongly Disagree
Please select:	◯	◯	◯	◯	◯

<b>

143. I value the ability to do the activities I want in VIRTUAL ENVIRONMENTS at any time I choose/when it suits me

	Strongly Agree	Agree	Neither agree or disagree	Disagree	Strongly Disagree
Please select:	○	○	○	○	○

144. I prefer to use VIRTUAL ENVIRONMENTS only when the people I interact with are online at the exact same time

	Strongly Agree	Agree	Neither agree or disagree	Disagree	Strongly disagree
Please select:	○	○	○	○	○

145. I value the ability to use VIRTUAL ENVIRONMENTS not just at home but when I am elsewhere too

	Strongly Agree	Agree	Neither agree or disagree	Disagree	Strongly Disagree
Please select:	○	○	○	○	○

146. I value the way VIRTUAL ENVIRONMENTS act as a kind of filter to guide me to useful information

	Strongly Agree	Agree	Neither agree or disagree	Disagree	Strongly Disagree
Please select:	○	○	○	○	○

147. I value it when there is a health professional contributing to VIRTUAL ENVIRONMENT use

	Strongly Agree	Agree	Neither agree or disagree	Disagree	Strongly Disagree
Please select:	○	○	○	○	○

148. I value it when there is a group facilitator/moderator overseeing VIRTUAL ENVIRONMENT use

	Strongly Agree	Agree	Neither agree or disagree	Disagree	Strongly Disagree
Please select:	○	○	○	○	○

149. VIRTUAL ENVIRONMENTS are an effective platform to record stories of chronic pain (yours and other people's)

	Strongly Agree	Agree	Neither agree or disagree	Disagree	Strongly Disagree
Please select:	○	○	○	○	○

150. I value being able to share my experiences through VIRTUAL ENVIRONMENTS

	Strongly Agree	Agree	Neither agree or disagree	Disagree	Strongly Disagree
Please select:	○	○	○	○	○

<b>

151. I value being able to find out about others' experiences through VIRTUAL ENVIRONMENTS

	Strongly Agree	Agree	Neither agree or disagree	Disagree	Strongly Disagree
Please select:	○	○	○	○	○

152. I use VIRTUAL ENVIRONMENTS differently depending on the stage of my condition

	Strongly Agree	Agree	Neither agree or disagree	Disagree	Strongly Disagree
Please select:	○	○	○	○	○

153. When my condition is severe or flared-up, I use VIRTUAL ENVIRONMENTS..

	Never	Almost never	Sometimes	Fairly often	Very often
Please select:	○	○	○	○	○

154. When my condition is stable, I use VIRTUAL ENVIRONMENTS..

	Never	Almost never	Sometimes	Fairly often	Very often
Please select:	○	○	○	○	○

<b>

155. Do you feel that your use of VIRTUAL ENVIRONMENTS has in any way helped your..

Note: You only need to respond to those items relevant to your situation

	Very much	Quite a bit	Somewhat	A little bit	Not at all
Ability to take in new information					
Ability to concentrate					
Ability to sleep					
Enjoyment of life					
Ability to participate in social activities					
Relationships with other people					
Family life					
Ability to perform your day-to-day activities					
Ability to do household chores					
Ability to work (include work at home)					
Emotional burden					
Anxiety					
Depression					
Ability to sit for more than 30 minutes					
Ability to stand for more than 30 minutes					
Ability to walk more than 1 mile (1.6 km)					

156. Please use this space if you would like to comment further about your use of VIRTUAL ENVIRONMENTS for your chronic pain self-management..

<b>

VIDEO SHARING SITES

The following questions relate specifically to **VIDEO SHARING SITES**

Description: Video Sharing Sites are platforms that enable the publishing and sharing of video content

Examples include but are not limited to: YouTube, Vimeo, etc

***157. In the last year, have you used VIDEO SHARING SITES when you go online for information, communication or interaction about your chronic pain?**

◯ Yes

◯ No

<b>

158. How valuable are VIDEO SHARING SITES for information, communication or interaction about your chronic pain?

◯ Not at all ◯ A little bit ◯ Somewhat ◯ Quite a bit ◯ Very much

159. How often do you use VIDEO SHARING SITES as part of your chronic pain self-management?

	At least daily	At least weekly	At least monthly	Less than once a month
Please select:	◯	◯	◯	◯

160. Please select the specific things you do on VIDEO SHARING SITES for your chronic pain self-management? (you can select more than one):

- ☐ Watch a video
- ☐ Post my own video
- ☐ Share a video
- ☐ Rate a video
- ☐ Comment on a video
- ☐ Subscribe to a channel

Other (please specify)

With your use of VIDEO SHARING SITES for chronic pain self-management in mind, please indicate your level of agreement with the following statements..

Note: If you feel a statement is **'NOT APPLICABLE'** to this platform, **LEAVE IT BLANK**

161. I prefer to control the amount and sorts of things that other people know about me on VIDEO SHARING SITES

	Strongly Agree	Agree	Neither agree or disagree	Disagree	Strongly Disagree
Please select:	◯	◯	◯	◯	◯

162. I prefer to know personal information about the people I interact with on VIDEO SHARING SITES

	Strongly Agree	Agree	Neither agree or disagree	Disagree	Strongly Disagree
Please select:	◯	◯	◯	◯	◯

<b>

163. I prefer to remain anonymous on VIDEO SHARING SITES

	Strongly Agree	Agree	Neither agree or disagree	Disagree	Strongly Disagree
Please select:	◯	◯	◯	◯	◯

164. I value the ability to do the activities I want on VIDEO SHARING SITES at any time I choose/when it suits me

	Strongly Agree	Agree	Neither agree or disagree	Disagree	Strongly Disagree
Please select:	◯	◯	◯	◯	◯

165. I prefer to use VIDEO SHARING SITES only when the people I interact with are online at the exact same time

	Strongly Agree	Agree	Neither agree or disagree	Disagree	Strongly disagree
Please select:	◯	◯	◯	◯	◯

166. I value the ability to use VIDEO SHARING SITES not just at home but when I am elsewhere too

	Strongly Agree	Agree	Neither agree or disagree	Disagree	Strongly Disagree
Please select:	◯	◯	◯	◯	◯

167. I value the way VIDEO SHARING SITES act as a kind of filter to guide me to useful information

	Strongly Agree	Agree	Neither agree or disagree	Disagree	Strongly Disagree
Please select:	◯	◯	◯	◯	◯

168. I value it when there is a health professional contributing to VIDEO SHARING SITE use

	Strongly Agree	Agree	Neither agree or disagree	Disagree	Strongly Disagree
Please select:	◯	◯	◯	◯	◯

169. I value it when there is a group facilitator/moderator overseeing VIDEO SHARING SITE use

	Strongly Agree	Agree	Neither agree or disagree	Disagree	Strongly Disagree
Please select:	◯	◯	◯	◯	◯

170. VIDEO SHARING SITES are an effective platform to record stories of chronic pain (yours and other people's)

	Strongly Agree	Agree	Neither agree or disagree	Disagree	Strongly Disagree
Please select:	◯	◯	◯	◯	◯

<b>

171. I value being able to share my experiences through VIDEO SHARING SITES

	Strongly Agree	Agree	Neither agree or disagree	Disagree	Strongly Disagree
Please select:	◯	◯	◯	◯	◯

172. I value being able to find out about others' experiences through VIDEO SHARING SITES

	Strongly Agree	Agree	Neither agree or disagree	Disagree	Strongly Disagree
Please select:	◯	◯	◯	◯	◯

173. I use VIDEO SHARING SITES differently depending on the stage of my condition

	Strongly Agree	Agree	Neither agree or disagree	Disagree	Strongly Disagree
Please select:	◯	◯	◯	◯	◯

174. When my condition is severe or flared-up, I use VIDEO SHARING SITES..

	Never	Almost never	Sometimes	Fairly often	Very often
Please select:	◯	◯	◯	◯	◯

175. When my condition is stable, I use VIDEO SHARING SITES..

	Never	Almost never	Sometimes	Fairly often	Very often
Please select:	◯	◯	◯	◯	◯

<b>

176. Do you feel that your use of VIDEO SHARING SITES has in any way helped your..

<u>Note:</u> You only need to respond to those items relevant to your situation

	Very much	Quite a bit	Somewhat	A little bit	Not at all
Ability to take in new information	◯	◯	◯	◯	◯
Ability to concentrate	◯	◯	◯	◯	◯
Ability to sleep	◯	◯	◯	◯	◯
Enjoyment of life	◯	◯	◯	◯	◯
Ability to participate in social activities	◯	◯	◯	◯	◯
Relationships with other people	◯	◯	◯	◯	◯
Family life	◯	◯	◯	◯	◯
Ability to perform your day-to-day activities	◯	◯	◯	◯	◯
Ability to do household chores	◯	◯	◯	◯	◯
Ability to work (include work at home)	◯	◯	◯	◯	◯
Emotional burden	◯	◯	◯	◯	◯
Anxiety	◯	◯	◯	◯	◯
Depression	◯	◯	◯	◯	◯
Ability to sit for more than 30 minutes	◯	◯	◯	◯	◯
Ability to stand for more than 30 minutes	◯	◯	◯	◯	◯
Ability to walk more than 1 mile (1.6 km)	◯	◯	◯	◯	◯

177. Please use this space if you would like to comment further about your use of VIDEO SHARING SITES for your chronic pain self-management..

<b>

PHOTO SHARING SITES

The following questions relate specifically to **PHOTO SHARING SITES**

Description: Photo Sharing Sites are platforms that enable the publishing and sharing of photo content

Examples include but are not limited to: Instagram, Flickr, etc

*** 178. In the last year, have you used PHOTO SHARING SITES when you go online for information, communication or interaction about your chronic pain?**

◯ Yes

◯ No

<b>

179. How valuable are PHOTO SHARING SITES for information, communication or interaction about your chronic pain?

◯ Not at all ◯ A little bit ◯ Somewhat ◯ Quite a bit ◯ Very much

180. How often do you use PHOTO SHARING SITES as part of your chronic pain self-management??

	At least daily	At least weekly	At least monthly	Less than once a month
Please select:	◯	◯	◯	◯

181. Please select the specific things you do on PHOTO SHARING SITES for your chronic pain self-management? (you can select more than one):

- ☐ Look at photos
- ☐ Post my own photos
- ☐ Share photos
- ☐ Rate/like a photo
- ☐ Tag a photo
- ☐ Comment on a photo
- ☐ Follow someone's photo streams

Other (please specify)

With your use of PHOTO SHARING SITES for chronic pain self-management in mind, please indicate your level of agreement with the following statements..

Note: If you feel a statement is **'NOT APPLICABLE'** to this platform, **LEAVE IT BLANK**

182. I prefer to control the amount and sorts of things that other people know about me on PHOTO SHARING SITES

	Strongly Agree	Agree	Neither agree or disagree	Disagree	Strongly Disagree
Please select:	◯	◯	◯	◯	◯

183. I prefer to know personal information about the people I interact with on PHOTO SHARING SITES

	Strongly Agree	Agree	Neither agree or disagree	Disagree	Strongly Disagree
Please select:	◯	◯	◯	◯	◯

<b>

184. I prefer to remain anonymous on PHOTO SHARING SITES

	Strongly Agree	Agree	Neither agree or disagree	Disagree	Strongly Disagree
Please select:	◯	◯	◯	◯	◯

185. I value the ability to do the activities I want on PHOTO SHARING SITES at any time I choose/when it suits me

	Strongly Agree	Agree	Neither agree or disagree	Disagree	Strongly Disagree
Please select:	◯	◯	◯	◯	◯

186. I prefer to use PHOTO SHARING SITES only when the people I interact with are online at the exact same time

	Strongly Agree	Agree	Neither agree or disagree	Disagree	Strongly disagree
Please select:	◯	◯	◯	◯	◯

187. I value the ability to use PHOTO SHARING SITES not just at home but when I am elsewhere too

	Strongly Agree	Agree	Neither agree or disagree	Disagree	Strongly Disagree
Please select:	◯	◯	◯	◯	◯

188. I value the way PHOTO SHARING SITES act as a kind of filter to guide me to useful information

	Strongly Agree	Agree	Neither agree or disagree	Disagree	Strongly Disagree
Please select:	◯	◯	◯	◯	◯

189. I value it when there is a health professional contributing to the PHOTO SHARING SITE use

	Strongly Agree	Agree	Neither agree or disagree	Disagree	Strongly Disagree
Please select:	◯	◯	◯	◯	◯

190. I value it when there is a group facilitator/moderator overseeing PHOTO SHARING SITES use

	Strongly Agree	Agree	Neither agree or disagree	Disagree	Strongly Disagree
Please select:	◯	◯	◯	◯	◯

191. PHOTO SHARING SITES are an effective platform to record stories of chronic pain (yours and other people's)

	Strongly Agree	Agree	Neither agree or disagree	Disagree	Strongly Disagree
Please select:	◯	◯	◯	◯	◯

<b>

192. I value being able to share my experiences through PHOTO SHARING SITES

	Strongly Agree	Agree	Neither agree or disagree	Disagree	Strongly Disagree
Please select:	◯	◯	◯	◯	◯

193. I value being able to find out about others' experiences through PHOTO SHARING SITES

	Strongly Agree	Agree	Neither agree or disagree	Disagree	Strongly Disagree
Please select:	◯	◯	◯	◯	◯

194. I use PHOTO SHARING SITES differently depending on the stage of my condition

	Strongly Agree	Agree	Neither agree or disagree	Disagree	Strongly Disagree
Please select:	◯	◯	◯	◯	◯

195. When my condition is severe or flared-up, I use PHOTO SHARING SITES..

	Never	Almost never	Sometimes	Fairly often	Very often
Please select:	◯	◯	◯	◯	◯

196. When my condition is stable, I use PHOTO SHARING SITES..

	Never	Almost never	Sometimes	Fairly often	Very often
Please select:	◯	◯	◯	◯	◯

<b>

197. Do you feel that your use of PHOTO SHARING SITES has in any way helped your..

<u>Note:</u> You only need to respond to those items relevant to your situation

	Very much	Quite a bit	Somewhat	A little bit	Not at all
Ability to take in new information	○	○	○	○	○
Ability to concentrate	○	○	○	○	○
Ability to sleep	○	○	○	○	○
Enjoyment of life	○	○	○	○	○
Ability to participate in social activities	○	○	○	○	○
Relationships with other people	○	○	○	○	○
Family life	○	○	○	○	○
Ability to perform your day-to-day activities	○	○	○	○	○
Ability to do household chores	○	○	○	○	○
Ability to work (include work at home)	○	○	○	○	○
Emotional burden	○	○	○	○	○
Anxiety	○	○	○	○	○
Depression	○	○	○	○	○
Ability to sit for more than 30 minutes	○	○	○	○	○
Ability to stand for more than 30 minutes	○	○	○	○	○
Ability to walk more than 1 mile (1.6 km)	○	○	○	○	○

198. Please use this space if you would like to comment further about your use of PHOTO SHARING SITES for your chronic pain self-management..

<b>

DISCUSSION FORUMS/MESSAGE BOARDS

The following questions relate specifically to **DISCUSSION FORUMS/MESSAGE BOARDS**

Description: Discussion Forums/Message Boards are earlier social web technologies that enable users to post messages and interact via message threads or topics (they are normally specific to an area of interest)

***199. In the last year, have you used DISCUSSION FORUMS/MESSAGE BOARDS when you go online for information, communication or interaction about your chronic pain?**

- ◯ Yes
- ◯ No

<b>

200. How valuable are DISCUSSION FORUMS/MESSAGE BOARDS for information, communication or interaction about your chronic pain?

◯ Not at all ◯ A little bit ◯ Somewhat ◯ Quite a bit ◯ Very much

201. How often do you use DISCUSSION FORUMS/MESSAGE BOARDS as part of your chronic pain self-management?

	At least daily	At least weekly	At least monthly	Less than once a month
Please select:	◯	◯	◯	◯

202. Please select the specific things you do on DISCUSSION FORUMS/MESSAGE BOARDS for your chronic pain self-management? (you can select more than one):

☐ Read posts
☐ Post yourself
☐ Comment on another person's post

Other (please specify)

With your use of DISCUSSION FORUMS/MESSAGE BOARDS for chronic pain self-management in mind, please indicate your level of agreement with the following statements..

Note: If you feel a statement is **'NOT APPLICABLE'** to this platform, **LEAVE IT BLANK**

203. I prefer to control the amount and sorts of things that other people know about me on DISCUSSION FORUMS/MESSAGE BOARDS

	Strongly Agree	Agree	Neither agree or disagree	Disagree	Strongly Disagree
Please select:	◯	◯	◯	◯	◯

204. I prefer to know personal information about the people I interact with on DISCUSSION FORUMS/MESSAGE BOARDS

	Strongly Agree	Agree	Neither agree or disagree	Disagree	Strongly Disagree
Please select:	◯	◯	◯	◯	◯

205. I prefer to remain anonymous on DISCUSSION FORUMS/MESSAGE BOARDS

	Strongly Agree	Agree	Neither agree or disagree	Disagree	Strongly Disagree
Please select:	◯	◯	◯	◯	◯

<b>

206. I value the ability to do the activities I want on DISCUSSION FORUMS/MESSAGE BOARDS any time I choose/when it suits me

	Strongly Agree	Agree	Neither agree or disagree	Disagree	Strongly Disagree
Please select:	◯	◯	◯	◯	◯

207. I prefer to use DISCUSSION FORUMS/MESSAGE BOARDS only when the people I interact with are online at the exact same time

	Strongly Agree	Agree	Neither agree or disagree	Disagree	Strongly disagree
Please select:	◯	◯	◯	◯	◯

208. I value the ability to use DISCUSSION FORUMS/MESSAGE BOARDS not just at home but when I am elsewhere too

	Strongly Agree	Agree	Neither agree or disagree	Disagree	Strongly Disagree
Please select:	◯	◯	◯	◯	◯

209. I value the way DISCUSSION FORUMS/MESSAGE BOARDS act as a kind of filter to guide me to useful information

	Strongly Agree	Agree	Neither agree or disagree	Disagree	Strongly Disagree
Please select:	◯	◯	◯	◯	◯

210. I value it when there is a health professional contributing to DISCUSSION FORUMS/MESSAGE BOARD use

	Strongly Agree	Agree	Neither agree or disagree	Disagree	Strongly Disagree
Please select:	◯	◯	◯	◯	◯

211. I value it when there is a group facilitator/moderator overseeing DISCUSSION FORUMS/MESSAGE BOARD use

	Strongly Agree	Agree	Neither agree or disagree	Disagree	Strongly Disagree
Please select:	◯	◯	◯	◯	◯

212. DISCUSSION FORUMS/MESSAGE BOARDS are an effective platform to record stories of chronic pain (yours and other people's)

	Strongly Agree	Agree	Neither agree or disagree	Disagree	Strongly Disagree
Please select:	◯	◯	◯	◯	◯

<b>

213. I value being able to share my experiences through DISCUSSION FORUMS/MESSAGE BOARDS

	Strongly Agree	Agree	Neither agree or disagree	Disagree	Strongly Disagree
Please select:	◯	◯	◯	◯	◯

214. I value being able to find out about others' experiences through DISCUSSION FORUMS/MESSAGE BOARDS

	Strongly Agree	Agree	Neither agree or disagree	Disagree	Strongly Disagree
Please select:	◯	◯	◯	◯	◯

215. I use DISCUSSION FORUMS/MESSAGE BOARDS differently depending on the stage of my condition

	Strongly Agree	Agree	Neither agree or disagree	Disagree	Strongly Disagree
Please select:	◯	◯	◯	◯	◯

216. When my condition is severe or flared-up, I use DISCUSSION FORUMS/MESSAGE BOARDS..

	Never	Almost never	Sometimes	Fairly often	Very often
Please select:	◯	◯	◯	◯	◯

217. When my condition is stable, I use DISCUSSION FORUMS/MESSAGE BOARDS..

	Never	Almost never	Sometimes	Fairly often	Very often
Please select:	◯	◯	◯	◯	◯

<b>

218. Do you feel that your use of DISCUSSION FORUMS/MESSAGE BOARDS has in any way helped your..

<u>Note:</u> You only need to respond to those items relevant to your situation

	Very much	Quite a bit	Somewhat	A little bit	Not at all
Ability to take in new information	○	○	○	○	○
Ability to concentrate	○	○	○	○	○
Ability to sleep	○	○	○	○	○
Enjoyment of life	○	○	○	○	○
Ability to participate in social activities	○	○	○	○	○
Relationships with other people	○	○	○	○	○
Family life	○	○	○	○	○
Ability to perform your day-to-day activities	○	○	○	○	○
Ability to do household chores	○	○	○	○	○
Ability to work (include work at home)	○	○	○	○	○
Emotional burden	○	○	○	○	○
Anxiety	○	○	○	○	○
Depression	○	○	○	○	○
Ability to sit for more than 30 minutes	○	○	○	○	○
Ability to stand for more than 30 minutes	○	○	○	○	○
Ability to walk more than 1 mile (1.6 km)	○	○	○	○	○

219. Please use this space if you would like to comment further about your use of DISCUSSION FORUMS/MESSAGE BOARDS for your chronic pain self-management..

<b>

CHAT ROOMS

The following questions relate specifically to **CHAT ROOMS**

Description: Chat Rooms are platforms that allow for synchronous communication between people, all online at the same time (they are normally specific to an area of interest)

*** 220. In the last year, have you used CHAT ROOMS when you go online for information, communication or interaction about your chronic pain?**

◯ Yes

◯ No

<b>

221. How valuable are CHAT ROOMS for information, communication or interaction about your chronic pain?

○ Not at all ○ A little bit ○ Somewhat ○ Quite a bit ○ Very much

222. How often do you use CHAT ROOMS as part of your chronic pain self-management?

	At least daily	At least weekly	At least monthly	Less than once a month
Please select:	○	○	○	○

223. Please select the specific things you do in CHAT ROOMS for your chronic pain self-management? (you can select more than one):

- ☐ Follow conversations
- ☐ Contribute to conversation
- ☐ Start your own conversation

Other (please specify)

With your use of CHAT ROOMS for chronic pain self-management in mind, please indicate your level of agreement with the following statements..

Note: If you feel a statement is **'NOT APPLICABLE'** to this platform, **LEAVE IT BLANK**

224. I prefer to control the amount and sorts of things that other people know about me in CHAT ROOMS

	Strongly Agree	Agree	Neither agree or disagree	Disagree	Strongly Disagree
Please select:	○	○	○	○	○

225. I prefer to know personal information about the people I interact with in CHAT ROOMS

	Strongly Agree	Agree	Neither agree or disagree	Disagree	Strongly Disagree
Please select:	○	○	○	○	○

226. I prefer to remain anonymous in CHAT ROOMS

	Strongly Agree	Agree	Neither agree or disagree	Disagree	Strongly Disagree
Please select:	○	○	○	○	○

<b>

227. I value the ability to do the activities I want in CHAT ROOMS at any time I choose/when it suits me

	Strongly Agree	Agree	Neither agree or disagree	Disagree	Strongly Disagree
Please select:	○	○	○	○	○

228. I prefer to use CHAT ROOMS only when the people I interact with are online at the exact same time

	Strongly Agree	Agree	Neither agree or disagree	Disagree	Strongly disagree
Please select:	○	○	○	○	○

229. I value the ability to use CHAT ROOMS not just at home but when I am elsewhere too

	Strongly Agree	Agree	Neither agree or disagree	Disagree	Strongly Disagree
Please select:	○	○	○	○	○

230. I value the way CHAT ROOMS act as a kind of filter to guide me to useful information

	Strongly Agree	Agree	Neither agree or disagree	Disagree	Strongly Disagree
Please select:	○	○	○	○	○

231. I value it when there is a health professional contributing to CHAT ROOM use

	Strongly Agree	Agree	Neither agree or disagree	Disagree	Strongly Disagree
Please select:	○	○	○	○	○

232. I value it when there is a group facilitator/moderator overseeing CHAT ROOM use

	Strongly Agree	Agree	Neither agree or disagree	Disagree	Strongly Disagree
Please select:	○	○	○	○	○

233. CHAT ROOMS are an effective platform to record stories of chronic pain (yours and other people's)

	Strongly Agree	Agree	Neither agree or disagree	Disagree	Strongly Disagree
Please select:	○	○	○	○	○

234. I value being able to share my experiences through CHAT ROOMS

	Strongly Agree	Agree	Neither agree or disagree	Disagree	Strongly Disagree
Please select:	○	○	○	○	○

<b>

235. I value being able to find out others' experiences through CHAT ROOMS

	Strongly Agree	Agree	Neither agree or disagree	Disagree	Strongly Disagree
Please select:	◯	◯	◯	◯	◯

236. I use CHAT ROOMS differently depending on the stage of my condition

	Strongly Agree	Agree	Neither agree or disagree	Disagree	Strongly Disagree
Please select:	◯	◯	◯	◯	◯

237. When my condition is severe or flared-up, I use CHAT ROOMS..

	Never	Almost never	Sometimes	Fairly often	Very often
Please select:	◯	◯	◯	◯	◯

238. When my condition is stable, I use CHAT ROOMS..

	Never	Almost never	Sometimes	Fairly often	Very often
Please select:	◯	◯	◯	◯	◯

<b>

239. Do you feel that your use of CHAT ROOMS has in any way helped your..

Note: You only need to respond to those items relevant to your situation

	Very much	Quite a bit	Somewhat	A little bit	Not at all
Ability to take in new information	◯	◯	◯	◯	◯
Ability to concentrate	◯	◯	◯	◯	◯
Ability to sleep	◯	◯	◯	◯	◯
Enjoyment of life	◯	◯	◯	◯	◯
Ability to participate in social activities	◯	◯	◯	◯	◯
Relationships with other people	◯	◯	◯	◯	◯
Family life	◯	◯	◯	◯	◯
Ability to perform your day-to-day activities	◯	◯	◯	◯	◯
Ability to do household chores	◯	◯	◯	◯	◯
Ability to work (include work at home)	◯	◯	◯	◯	◯
Emotional burden	◯	◯	◯	◯	◯
Anxiety	◯	◯	◯	◯	◯
Depression	◯	◯	◯	◯	◯
Ability to sit for more than 30 minutes	◯	◯	◯	◯	◯
Ability to stand for more than 30 minutes	◯	◯	◯	◯	◯
Ability to walk more than 1 mile (1.6 km)	◯	◯	◯	◯	◯

240. Please use this space if you would like to comment further about your use of CHAT ROOMS for your chronic pain self-management..

<b>

END OF SURVEY

Congratulations! You have completed our survey. Thank you for taking the time. Your responses will be of great insight
