# Supplementary material for: Therapeutic Affordances of Social Media: Emergent Themes From a Global Online Survey of People With Chronic Pain
Source: J Med Internet Res. 2014 Dec 22;16(12):e284. doi: 10.2196/jmir.3494 (PMC4285739; doi:10.2196/jmir.3494)
Supplement: Supplementary file 2 [file jmir_v16i12e284_app2.pdf]

Appendix 2. Visualization of full data set

Positive Quotes

Platform/Affordance	Self-presentation	Connection	Exploration	Narration	Adaptation
**SNS (N = 58 responses), of 189 to use**<br><br>**31% of SNS participants**		"I feel valued sharing my resources with others" (resp 79)<br><br>"Without social networking I would feel far more alone when my conditions are bad.." (resp 46)<br><br>"Useful for information from websites not based in my country.." (resp 70)<br><br>"..pain is isolating in real life and social networking can help to reduce the amount of isolation" (resp 56)<br><br>"..[SNS] is often putting patients in touch with treating practitioners around the world" (resp 79)<br><br>"We are able to help each other with practical advice	"I much prefer a medically knowledgeable, balanced platform such as a monitored [one] where good, sensible advice and help is offered" (resp 40)<br><br>"Emotionally it is enormously helpful to find others with shared situations" (resp 40)<br><br>"..helps when looking at new information or needing emotional support" (resp 12)<br><br>"..find relevant research and papers" (resp 83)<br><br>"Finding other people with exactly the same strange symptoms is a great emotional relief"	"I like to share my experience with other sufferers. The more we learn the easier it is to cope with our experiences" (resp 103)<br><br>"..networking sites make me feel useful as I can share my stories with newly diagnosed people" (resp 185)<br><br>"It is very helpful to share stories, symptoms, and problems with other sufferers" (resp 223)<br><br>"I find it helpful to know how other people manage there own pain" (resp 191)<br><br>"It's good to hear stories similar to my own to know that I'm not alone" (rep 180)<br><br>"keen reader of others' experiences, thoughts etc"	"Social networking is vital in keeping me connected to my family friends and outside world when I'm sick or in hospital" (resp 185)<br><br>"I find scrolling sites like tumblr and Instagram can be a good distraction when in a lot of pain" (resp 185)<br><br>"It can also provide entertainment on days when I spend a lot of time in bed sick" (resp 185)<br><br>"It is a good way to pass time when I cannot sleep at night" (resp 186)<br><br>"Social network sites allow me to interact with others on my own terms" (resp 184)<br><br>"I use different social

about drug side effects etc (resp 83)”<br><br>“I try as much as possible to share back the information and support I have gained from it “(resp 154)<br><br>“I have made some amazing friends with chronic pain on social network sites - some I have met and some I have not but I feel less alone” (resp 174)<br><br>“I focus on sharing what knowledge or information I have in other areas, this mitigates the constant feeling of failure that comes along with ill health and the inability to be more active” (resp 177)<br><br>“It is a good format to use for information sharing and tips..” (resp 186)<br><br>“Helps to know I’m not alone” (resp 209)<br><br>“..only a person with similar problems really	(resp 83)<br><br>“It would be much better if there were a proper dietician moderating it..” (resp 154)<br><br>“I get a lot of information on them” (resp 190)<br><br>“..I open [SNS] and I come to realize" It's not just me" I'm not crazy/mad/insane/it's just in my head” (resp 205)<br><br>“[SNS] make symptoms a bit more easier to understand” (resp 205)<br><br>“Have particularly used social networking to learn tips about managing/avoiding pain” (resp 112)<br><br>“I have found the information..going through pain management products or natural nutrition very valuable” (resp 109)	(resp 165)<br><br>“..to hear about different treatments and people's experiences of them” (resp 112)<br><br>“having people who can make comments and talk about their stories give me more information about what other people are dealing with” (resp 32)<br><br>“Social networking gives CP patients a platform to share their story” (resp 31)<br><br>“Interesting to hear about treatments and help programmes” (resp 7)	network sites in different ways” (resp 41)

gets what your going through” (resp 201)

“I find general support from others with the same condition on these sites has stopped my suicide attempts” (resp 217)

“Having immediate access to information & people is enlightening” (resp 206)

“Social network sites have allowed me to have a social life..when the pain is bad, which is frequent, I cannot leave my house and spend time with friends” (resp 196)

“Talking to people who understand is great” (resp 180)

“I live in a regional area and helpful Dr. services are not available! I'm discovering the positives of using the sites” (resp 164)

“It has helped me realise I'm not alone” (resp 164)

“They help to mitigate the

“It is great for advice..” (resp 78)

“The beauty of social media is that there is no censor or control so the right information gets through” (resp 78)

“It's now up to people to go out and find the right information for themselves..” (resp 78)

“I get information from it how to live, how to prevent further pain..” (resp 13)

“Learned good tips on self-management” (resp 1)

isolation” (resp 163)

“..[social network sites] actually saved my life. The people I connected with were other with MS…we all support each other..” (resp 140)

“..we all support each other during our tough times and still communicate even during our good days” (resp 140)

“It is extremely good to talk with others who understand what you are experiencing as then you do not feel alone.. only other sufferers truly understand” (resp 140)

“[Social network sites] can be an excellent platform to discuss my chronic pain with other people” (resp 84)

“Personally I have found being able to meet other people of a similar age dealing with the same issues as me invaluable. In fact it has been a lifeline.”

(resp 80)

“..people all over the world can communicate and support others in their same situation” (resp 78)

“Sharing alternate remedies is crucial as western medicine does not give us this information” (resp 78)

“I have friends worldwide who know what I am experiencing..” (resp 71)

“The friendships and information I've gained from those have been life saving - probably in the literal sense” (resp 71)

“When first diagnosed I had never heard of CRPS - now I have friends worldwide.. they are there day or night if I need” (resp 71)

The exchange of information available is invaluable” (resp 38)

“social network sites are

		excellent for getting in touch with other people who have the disease..” (resp 10)<br><br>“..they make you feel like you are not alone in your pain” (resp 10)<br><br>“Social network sites help lessen my feeling of isolation” (resp 7)<br><br>“..helps when..needing emotional support” (resp 12)		
**DISCUSSION FORUMS (N = 18 responses), of 86 to use**<br><br>**21% of DF participants**	“I find these boards are more intimate if I can remain anonymous to family and friends but known to others on the board” (resp 176)<br><br>“The discussion forum we use is a private support group, so only other sufferers of my condition are able to participate” (resp 204)	“The exchange of information on some message boards.. is invaluable” (resp 31)<br><br>“..give and return information” (resp 227)<br><br>“..positive to see you are not alone” (resp 6)	“I have found a couple of particular knowledgeable sites invaluable..” (resp 229)<br><br>“Practical tips, medication, treatment recommendations have often lead me to helpful sites” (resp 190)<br><br>“..one becomes discriminating with the types of boards and if the moderator is a good one, this sets the tone of the board and generally adds to the value of the information (resp 190)	“..people who go through all (and more) I go through” (resp 207)<br><br>“I mostly go on discussion boards to read people's experiences” (resp 208)<br><br>“Many of my own experiences were also shared by others. Often it is quite validating to see others have been thru the same things I have” (resp 190)

			“I have been a member of a forum where a pain management specialist was a regular contributor...and his contributions were invaluable” (resp 191)<br><br>“Forums have helped me when I need info about side affects of drugs relating to my condition..” (resp 221)<br><br>“Forums have helped.. in how other fellow sufferers cope” (resp 221)<br><br>“Forums are where I start research most of the time” (resp 27)<br><br>“The discussion forum we use..is strongly monitored” (resp 204)	
**BLOGS (N = 11 responses), of 88 to use**<br><br>**13% of BLOG participants**	“..writing a (decent-ish) blog can allow you to be the person you 'were' to an extent” (resp 229)	“Being able to communicate with people can be up lifting and cathartic” (resp 229)<br><br>“I feel I am not alone. My invisible friend I can share with. Who understands	“use blogs so as I can learn as much as I can about my condition. Knowledge is power, etc” (resp 227)<br><br>“Value depends on quality of information”	“Sometimes reading other peoples journeys is useful..” (resp 209)<br><br>“I write reflective and creative pieces about my journey in general..often I won't even discuss the

	me” (resp 146)	(resp 190)<br><br>“I use them for information on the management of pain” (resp 10)<br><br>“The blogs of health researchers and professionals are very important tools for finding new information, current research etc” (resp 217)	physical side of it, but rather the lessons learned” (resp 189)<br><br>“It just helps to know there are other people in the same boat who work hard at their disorder and succeed. It gives me hope and patience to keep trying” (resp 212)
**WIKIS (N = 10 responses), of 74 to use**<br><br>**14% of WIKI participants**		“I often refer to anatomical diagrams, medical and scientific info wikipedia provides” (resp 229)<br><br>“..use mostly for education and fact-finding” (resp 222)<br><br>“I just use wikis to read up on my conditions and see if any new relevant research has been linked to it” (resp 215)<br><br>“..wikis don't do anything other than give me information the rest is up to my actions” (resp 209)	

			“Gives me more information about a specific condition or more things to discuss with my doctor..” (resp 200)<br><br>“Wikis are good to research things..” (resp 66)<br><br>“Wikis have been most useful as a crude starting point..” (resp 217)<br><br>“To get information” (resp 10)		
**VIDEO SHARING SITES (N = 7 responses), of 60 to use**<br><br>**12% of VSS participants**		“..are mostly helpful due to the "you are not alone" value” (resp 215)<br><br>“Fellow severe M.E. sufferers also help me feel less isolated..given my lack of social connection” (resp 190)<br><br>“International M.E. Specialists whom I would NEVER have access to otherwise..” (resp 190)	“They have definitely helped my understanding of nerve pain and pain in general. I have grasped anatomical and medical info much easier with YouTube” (resp 229)<br><br>“I have watched yoga and tai chi videos to learn about exercises” (resp 206)<br><br>“TED talks have been invaluable ways of	“Seeing others who are like me, is a HUGE comfort..” (resp 190)	“I am severely affected at present. I cannot speak, or tolerate the stimulation of people being around me.. I am bedridden and need to be in a very low stimulus environment.. but youtube is ok for me with care” (resp 190)<br><br>“I use comedic and inspirational videos during flare-ups to assist with state of mind, depression etc” (resp 217)

			sourcing new information” (resp 217)		“It's nice to be able to learn even when I can't read or sit up” (resp 27)
**MICROBLOGS (N =6 responses), of 29 to use**<br><br>**21% of MICROBLOGS participants**		“Micro blogging using twitter has helped me feel less isolated” (resp 221)	“As always information, information, information” (resp 227)<br><br>“..of most value to alert me to new research papers, conferences, clinical trials, new treatment protocols etc worthy of further investigation” (resp 190)<br><br>“..to get information” (resp 10)<br><br>“..for finding inspirational quotes that sometimes help” (resp 8)	“It's great to be able to follow other young women with chronic conditions on Tumblr…it’sgreat to laugh about our situation..” (resp 189)	
**PHOTO SHARING SITES (N = 5 responses), of 18 to use**<br><br>**28% of PSS participants**		“..It's reassuring to have someone who can say - 'look, this happened to me too'” (resp 191)		“I will post a photo on Instagram if I am in hospital and people want to know how I am going..”(resp 189)<br><br>“I have also used photos to show people just what having CRPS looks like” (resp 191)<br><br>“Photos have been a great interactive tool. Not just for sharing as awareness for my condition but sharing of non	“I will post a photo on Instagram if I am in hospital and people want to know how I am going but I am too sick to tell them” (resp 189)<br><br>“I've found Instagram a great way of being able to update friends and family while I'm in the hospital, without having to spend a lot of energy typing up a

				relevant pics eg. humourr/inspiration” (resp 221)<br><br>“I can take a snap of wherever I am, or what's happening..it updates everyone quickly and efficiently” (resp 203)<br><br>“I've found photos a really powerful tool for other "healthy" people to understand what it's like to be chronically ill” (resp 203)<br><br>“Sometimes, seeing a photo of a certain procedure is more powerful than reading a long update about what's happening in my life” (resp 203)	status update” (resp 203)
**CHAT ROOMS (N = 3 responses), of 11 to use**<br><br>**27% of participants**			“I only use a moderated chat room..” (resp 189)<br><br>“I selectively filter through information.. I look for information around the management of pain and adapting in certain situations” (resp 51)		“Good for short-term emergency reassurance when things are really bad..” (resp 125)
**VIRTUAL ENVIRONMENTS**	“This is an excellent medium for me to be able to control my social interaction..” (resp 120)	“..to learn new skills at my own pace” (resp 120)		“..a means of creative expression.. (resp 120)	

**(N = 2 responses), of 7 to use**		"I have also the possibility of support group interaction without getting out of bed. It is of immense value to me" (resp 120)			
**29% of participants TAGGING/AGGREGATION SITES (N = 2 responses), of 12 to use**			"..use Evernote to tag & clip and websites and other useful info" (resp 222)		
**17% of participants**					

Negative Quotes

Platform/Affordance	Self-presentation	Connection	Exploration	Narration	Adaptation
**SNS (N = 58)**	"My fears around disclosure and how it impacts psychologically physically and emotionally is enough to prevent me from disclosing in the social media" (resp 64)<br><br>"I do not want to disclose my personal and painful journey via a	"Unmoderated sites are potentially dangerous, unqualified ppl routinely diagnose, recommend treatment, use research findings inappropriately, misinform other ppl who may not know any better" (resp 8)	"Have to be selective - some sites focus on positive constructive stuff and that's helpful, others end up with "poor me" encourage self-pity and negativity and need to stay away from those. The same site can	"I do find many social network chronic pain sites regular participants too needy. I can't abide the constant call for, supply of prayers and the desperation for others to feel/understand/get their pain" (resp 40)	"I am newly diagnosed and have yet to come to terms with this..I become very distressed when I think/write/talk about my condition and the impact it has had on my life" (resp 64)

	social network site for it to be highlighted by others and "used" as a way to finish me in my job" (resp 64)	"Social networking can sometimes have a negative impact ie withdrawal from real life" (resp 56)<br><br>"Frustrating and at times more painful trying to explain to strangers" (resp 124)	change from one to the other over time too" (resp 170)<br><br>"Increase depression levels when you see how much fun your peers are having participating in activities" (resp 84)	"..sites become a platform for particular ppl to use to verbalise complaints and negativity, without any constructive discourse" (resp 8)<br><br>"It can be even more depressing knowing there are billions suffering with no help!" (resp 124)<br><br>"Unfortunately it can often feel that when people read your posts in regard to chronic pain, it can create speculation about the condition..it can make me feel, especially being a young male, that I am just a whinger and need to toughen up" (resp 84)<br><br>"I do not feel able to share my experience as yet as I become very distressed when I think/write/talk about my condition.." (resp 47)
**DISCUSSION FORUMS (N = 13)**	"…I see individuals masquerading as members of the medical profession.." (resp 191)	"I see individuals masquerading as members of the medical profession and often misleading pain patients" (resp 191)		

**BLOGS (N = 11)**	“Some blogs can be unhelpful, depressing, misleading” (resp 190)	“Sometimes reading other peoples journeys is useful but I do not like to immerse myself in too many down stories as it makes me worse so I am careful about what I read” (resp 209)<br><br>“I find most blogs very distressing as I "see" how my future may progress” (resp 51)
**WIKIS (N = 10)**	“Wikis are not always particularly useful, as entries tend to be very general and not always up to date” (resp 190)<br><br>“..can be misleading depending on who has written the article..” (resp 66)	
**VIDEO SHARING SITES (N = 7)**		
**MICROBLOGS (N =6)**		
**PHOTO SHARING SITES (N = 5)**		
**CHAT ROOMS (N = 3)**	“Seem to be most prone to bias..so bad information doesn't get corrected” (resp 125)	“I find it very distressing reading about the deterioration and "loss" of someone's purpose/function in

		life" (resp 51)
**VIRTUAL ENVIRONMENTS (N = 2)**		
**TAGGING/AGGREGATION SITES (N = 2)**		
